# Biochemical Characterization of Multimodular Xylanolytic Carbohydrate Esterases from the Marine Bacterium *Flavimarina* sp. Hel_I_48

**DOI:** 10.1002/cbic.202500058

**Published:** 2025-04-08

**Authors:** Michelle Teune, Thorben Döhler, Daniel Bartosik, Thomas Schweder, Uwe T. Bornscheuer

**Affiliations:** ^1^ Department of Biotechnology and Enzyme Catalysis Institute of Biochemistry University of Greifswald Felix‐Hausdorff‐Straße 4 17489 Greifswald Germany; ^2^ Department of Pharmaceutical Biotechnology Institute of Pharmacy University of Greifswald Felix‐Hausdorff‐Straße 3 17489 Greifswald Germany

**Keywords:** biocatalysis, carbohydrate active enzymes, carbohydrates, multidomain enzymes, xylans

## Abstract

Carbohydrate‐active enzymes (CAZymes) are critical for sustainable biomass utilization due to their ability to degrade complex polysaccharides. Frequently, a multimodularity can be observed combining several CAZyme domains and activities in close proximity which can benefit this degradation process. In this study, three multimodular xylanolytic carbohydrate esterases (CEs), named Fl6, Fll1, and Fll4, originating from *Flavimarina* sp. Hel_I_48 that represent a novel arrangement of catalytic and/or binding domains, are investigated. While Fl6 acts as a glucuronyl esterase, it also contains a carbohydrate binding module which is normally associated with xylanase activity. Fll1 combines xylosidase with acetylxylan esterase (AXE) activity mediated by a CE3 domain. The third enzyme, Fll4, is the first enzyme that comprises three distinct CE domains and shows bifunctional activity as an AXE and a feruloyl esterase (FAE). Investigation of the single domains reveals that the CE6 domain of Fll4 mediates its AXE activity while one of the putative CE1 domains, CE1a, mediates the FAE activity. This investigation of multimodularity of marine CAZymes not only enhances our understanding of these enzymes but may provide a promising route toward more efficient algal biomass utilization for biotechnological applications.

## Graphical abstract

Multimodular carbohydrate‐active enzymes often combine different catalytic activities for efficient polysaccharide degradation. In this work, the structural and functional characterization of three multimodular xylan‐targeting esterases, Fl6, Fll1 and Fll4 from *Flavimarina* sp. Hel_I_48, are presented. Each protein shows novelty in its architecture, with particular emphasis on the enzyme Fll4, which consists of three esterase domains, resulting in bifunctional esterase activity.

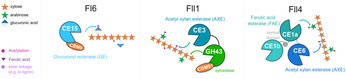

## 1 Introduction

Modern biotechnological research has made the sustainable manufacturing of chemicals, pharmaceuticals, and other high‐value products a major objective in response to growing environmental concerns and depleting petrochemical supplies. Exploiting renewable resources and developing enzyme‐based processes as a sustainable route toward the extraction of valuable components are key elements of these efforts. Among natural resources, marine algae (including seaweeds and microalgae) represent promising and diverse biomass feedstocks for the production of biofuels, food supplements, and platform chemicals, as they have high growth rates, do not compete with agricultural land, and contain a variety of valuable ingredients.^[^ ^1^, ^2^, ^3^ ^]^

Polysaccharides account for a major fraction of algal biomass, comprising alginate, carrageenan, laminarin, ulvan, and xylan—versatile polymers with applications ranging from food additives to pharmaceutical excipients.^[^ ^4^, ^5^, ^6^ ^]^ Xylan is the major hemicellulose in many terrestrial plants (e.g., hardwoods and cereal grains), but is also found in red and green algae, often exhibiting more complex side‐chain decorations and substitutions than its terrestrial counterparts.^[^ ^7^, ^8^ ^]^ Using efficient enzymatic depolymerization strategies, xylans can be hydrolyzed to fermentable sugars. In nature, xylanases are frequently found in marine microorganisms utilizing algal polysaccharides as carbon sources. Marine bacteria specialized in algal polysaccharide degradation most often belong to the *Bacteroidota* phylum.^[^ ^9^ ^]^ The corresponding genes for a variety of carbohydrate active enzymes (CAZymes), as well as other proteins like transport proteins or regulators, are often encoded in gene clusters, so‐called polysaccharide utilization loci (PULs) that allow the bacteria a highly specific and efficient substrate targeting.^[^ ^10^, ^11^, ^12^ ^]^

However, the presence of various modifications occurring in xylans, including ester‐linked substituents (e.g., ferulic acid, acetyl groups, or methyl glucuronic acids), complicates the direct action of glycoside hydrolases (GHs) like xylanases on the polysaccharide backbone. Consequently, xylanolytic carbohydrate esterases (CEs) are essential to remove these decorations to make the xylan backbone more accessible.^[^ ^13^, ^14^, ^15^, ^16^ ^]^ Xylanolytic feruloyl esterases (FAEs) naturally cleave linkages between arabinofuranoside side chains of arabinoxylans and ferulic or *p*‐coumaric acids that can be involved in lignin linkage or crosslinking of hemicelluloses, thereby increasing plant cell wall recalcitrance.^[^ ^14^, ^17^, ^18^, ^19^ ^]^ As phenolic acids are valuable components which can be used pharmaceutically, for example, as antioxidants, their recovery utilizing biomass could be crucial for future demands. Acetylxylan esterases (AXEs) catalyze the deacetylation of the xylan backbone where acetyl groups are found at the O–2 or O–3 position of the xylose residues. These decorations often serve as a defense mechanism while their removal results in a more efficient polysaccharide processing by GHs. AXEs can be found in many CE families like CE1, CE5, and CE6.^[^ ^20^, ^21^ ^]^

Glucuronoyl esterases (GEs) are the only enzymes known so far that can hydrolyze the ester bond between 4‐O‐methyl‐d‐glucuronic acid mainly present in glucuronoxylans and lignin which are often found in hardwood‐like beechwood.^[^ ^16^ ^]^ GEs can be exclusively found in CE family 15.^[^ ^22^ ^]^

A hallmark of many CAZymes is the presence of multiple domains (i.e., multimodular or multidomain enzymes). Such architectures may include two or more catalytic domains (e.g., different esterases) or combinations of catalytic domains with noncatalytic modules such as carbohydrate‐binding modules (CBMs). These complex enzyme architectures are likely evolutionary driven by the need to degrade more complex polysaccharides. As it is assumed that marine polysaccharides, which remain largely unexplored to date, demonstrate significantly greater complexity compared to their terrestrial counterparts, it is reasonable that multimodularity is observed frequently in the marine environment.^[^ ^23^, ^24^ ^]^

In this study, we therefore investigated three multimodular esterases from the marine bacterium *Flavimarina* sp. Hel_I_48, which were previously introduced as part of two distinct xylan PULs that presumably target arabinoxylan and glucuronoxylan. The esterase Fl6 is encoded within PULI, which has been shown to target glucuronoxylans such as beechwood xylan. Annotated as a CE15 esterase, Fl6 was confirmed to exhibit GE activity, consistent with the proposed glucuronoxylan specificity of PULI.^[^ ^11^ ^]^ This PUL also encodes another esterase previously annotated as a member of the CE6 family.^[^ ^11^ ^]^ PULII encodes for several enzymes that seem to be active on arabinoxylans. Among them are the esterases Fll1 and Fll4. While no activity could be detected for Fll1, Fll4 demonstrated both FAE and AXE activity.^[^ ^11^ ^]^ Here, we identified several new activities of Fll1 and Fll4 and revealed that Fll4 indeed consists of three distinct CE domains. Separation and investigation of the individual domains revealed that the CE6 domain mediates AXE activity, while one of the annotated CE1 domains is linked to FAE activity.

## 2 Results and Discussion

### 2.1 Xylanolytic CEs of *Flavimarina* sp. Hel_I_48 Consisting of Several Domains for Carbohydrate Degradation

**Figure 1:**
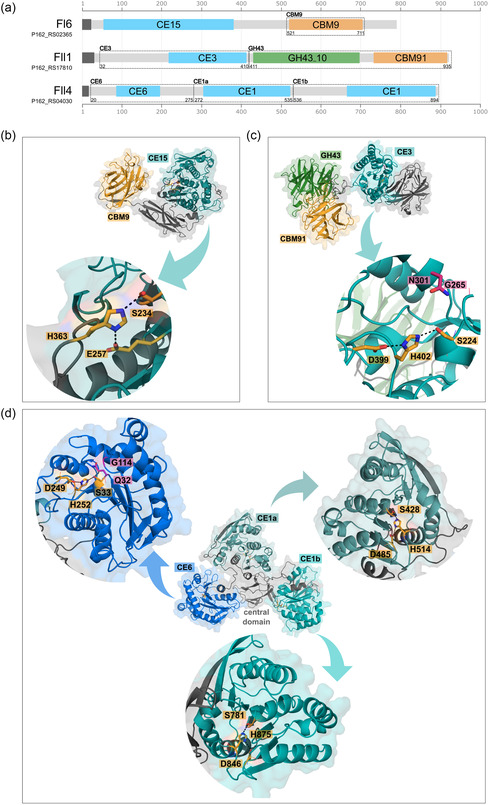
Domain architecture and AlphaFold3 models of the multimodular CEs Fl6, Fll1, and Fll4. a) Annotated domains using dcCAN‐HMMdb_v12. CE domains (shades of blue), GH domains (green), and CBMs (orange) are highlighted. The parts of the individually expressed domains are marked by dashed lines including the first and last amino acids of each domain (see Table S1, Supporting Information). AlphaFold3 models of the full‐length enzymes b) Fl6, c) Fll1, and d) Fll4. The CE, GH, and CBM domains are colored as described. Active sites of each CE domain are represented in a close‐up (blue arrows) with catalytic residue side chains shown as orange sticks. The oxyanion hole residue side chains are shown in pink when known. Dashed lines indicate the hydrogen bonds between the catalytic triad Ser‐His‐Asp residues.

In previous studies, the enzymes Fl6 (gene locus tag: P162_RS02365), Fll1 (P162_RS17810), and Fll4 (P162_RS04030) were described for the first time as part of two xylan‐responsive PULs in *Flavimarina* sp. Hel_I_48.^[^ ^11^ ^]^ While Fl6 was classified as a member of the CE15 family and the first domain of Fll4 as a part of family CE6, the annotation during these studies could not provide a clear assignment for the esterase domain of Fll1 and two out of three domains of Fll4. Thanks to ongoing database updates, a reannotation of the respective enzymes using different tools revealed that Fll1 is a putative CE3, while the second and third CE domains of Fll4 both were annotated as members of the CE1 family (**Figure** **1**a and S1, Supporting Information). Prediction of the protein structures using AlphaFold3^[^ ^25^ ^]^ was performed to compare the enzymes structural features to their respective CE family, partially confirming their sequence‐based annotation (Figure 1a–c). The confidence score of all predicted models (Figure S2a, S3a, and S4a, Supporting Information) shows an overall high confidence, especially in regions of secondary structures, but also the linker regions between domains seem to be predicted with high confidence levels. Fl6 as a part of the CE15 family, which is exclusively found to be GEs according to the CAZy database,^[^ ^22^ ^]^ shows a typical α/β‐hydrolase fold (Figure 1b) involving a three‐layer αβα‐sandwich fold with a high similarity (RMSD: 1.133) to the previously described crystal structure of a CE15 from *Myceliophthora thermophila*.^[^ ^26^ ^]^ The residues involved in the formation of the α‐helices and β‐sheets tend to be highly conserved according to ConSurf analyses (Figure S2b, Supporting Information).^[^ ^27^ ^]^ Electrostatic analyses of Fl6 show a high negative potential on one side of the protein (Figure S2c, Supporting Information). The other side, where the active site of the CE15 domain is located, suggests an overall slightly negative protein surface, except for the active site itself. Next to the catalytic serine S234, the oxyanion hole is located, indicated by a pocket with positive electrostatic potential for proper coordination of the oxyanion formed during catalysis. This observation can be made for all esterase domains (Figure S3d and S4d, Supporting Information). Besides the CE15 domain, Fl6 consists of a CBM9 domain which is C‐terminally attached to the esterase domain (Figure 1a,b). Until now, it was assumed that these domains are solely associated with xylanases.^[^ ^22^ ^]^ Although one example is known^[^ ^28^ ^]^ in which an enzyme contains a GH10 domain, a CE15 domain and three CBM9 domains, the Fl6 seems to be the first CE15 member which is associated with this domain without the additional contribution of a xylanase domain. The CBM9 family, which mostly occurs as a tandem, is known to interact with cellulose, xylan, and mannan, even though cellulose binding tends to be the strongest.^[^ ^28^, ^29^ ^]^ Additionally, binding to xyloglucan and xyloglucooligosaccharides was confirmed for the aforementioned GH10‐CE15 multidomain protein, suggesting a potential synergistic purpose of the binding domain to bind products of the endolytic xylanase for further degradation by the GE.^[^ ^28^ ^]^ For Fl6_CBM9, no binding of different xylan and xyloglucan polysaccharides could be observed using differential scanning fluorimetry (DSF) (Figure S5, Supporting Information). The identification of homologues of the esterase domain Fl6_CE15 using experimentally determined structures from the protein data bank (PDB) revealed several enzymes of the CE15 family (Figure S6, Supporting Information). All members show sequence similarity with Fl6_CE15 ranging from ≈27 to 56% (Figure S6b, Supporting Information). While the members which are showing moderate similarity like 8TSE (27.42%), also show fewer structural similarity (RMSD 1.829), highly similar sequences like 8TRX (55.56%), a fungal CE15 from rumen fungus *Piromyces rhizinflatus*,^[^ ^30^ ^]^ also show high structural similarity (RMSD 0.644) (Figure S6c, Supporting Information). Interestingly, both enzymes, Fl6 and 8TRX, miss an arginine residue which is reported to be highly conserved in CE15 enzymes and is contributing to the oxyanion hole formation.^[^ ^31^ ^]^

The predicted structure of the esterase domain of Fll1 (Figure 1c) as a member of the CE3 family shows a typical SGNH hydrolase fold, which appears in many CE families and targets several carbohydrate substrates.^[^ ^32^ ^]^ SGNH is referring to the conserved residues serine, glycine, asparagine, and histidine which can be found in the active site of these esterases just like in Fll1 (Figure 1c closeup). As confirmed by the conservation scores of Fll1_CE3 (Figure S3b, Supporting Information) the motifs around these residues are the most conserved ones in SGNH hydrolases. Besides the annotated CE3 domain, Fll1 contains a GH43_10 and a CBM91 domain which are both associated with xylosidase and arabinosidase activity. As the enzyme could not be expressed as a full‐length enzyme, the GH and CE domains have been characterized separately previously, revealing a xylosidase activity of the GH43 domain toward arabinoxylans.^[^ ^11^ ^]^ Sequence alignments of Fll1_CE3 with representatives of the CE3 family^[^ ^22^ ^]^ and homologous sequences identified from the PDB using basic local alignment search tool (BLAST) were performed (Figure S7a, Supporting Information). While all characterized CE3 members show little‐to‐no similarity to Fll1, high similarity can be observed for Fll1_CE3 with 7TOG and 7TOJ (64.96% and 57.02%). These enzymes are described to be members of a so far unclassified CE family acting as AXEs.^[^ ^33^ ^]^ It can be assumed that Fll1_CE3 could as well not belong to the CE3 family, as suggested by its annotation (Figure S1, Supporting Information), but rather belong to this new CE family.

Fll4 shows three distinct esterase domains which are arranged around an unclassified fourth domain in the center (Figure 1d). It is the first described enzyme consisting of three distinct esterase domains and showing this unique orientation of the domains. In the center, another domain can be found which cannot be clearly assigned, therefore it is named central domain in the following. Using dbCAN_sub, it was identified as a CBM48 domain (Figure S1, Supporting Information). However, since no other tools confirmed this annotation, it should be regarded with caution. This is especially true given that, according to the AlphaFold model, the domain does not exhibit a classic, CBM‐typical β‐sandwich fold. The three esterase domains of FII4 were annotated as members of the CE6 and CE1 family. As the second and third domain putatively both belong to family CE1, the second one is subsequently named CE1a and the third one CE1b. Like the CE3 family, the CE6 family shows the typical SGNH fold. As previously observed for CE6 enzymes, the asparagine in the SGNH motif, which is located in the oxyanion hole, can be functionally replaced by a glutamine.^[^ ^34^ ^]^ This can also be seen in the active site of Fll4_CE6 (Figure 1d). The CE6 family exclusively comprises AXE activity, which was also previously observed for full‐length Fll4.^[^ ^11^ ^]^ Besides the CE6 domain, two more CE domains can be found in Fll4. Both domains, again, are predicted to show a typical αβα‐sandwich fold typical for this family. The CE1 family is the largest CE family, acts through the conventional catalytic Ser‐His‐Asp/Glu triad, and it encompasses many different functions like AXE and FAE activity. Moreover, the family also contains enzymes that were observed to have transferase activity.^[^ ^22^, ^34^ ^]^

Sequence alignments (Figure S8 and S10a,b, Supporting Information) of the domains of Fll4 with representatives of their respective CE family and homologous sequences from the PDB were performed using BLAST. Additionally, we identified proteins from a PUL in the gut bacterium *Dysgonomonas mossii* (*Dm*) which show a highly similar domain architecture as Fll4. For the putative CE1 domains Fll4_CE1a and Fll4_CE1b, the alignment with characterized CE1 family members^[^ ^22^ ^]^ showed little‐to‐no similarity (Figure S8a, Supporting Information, yellow box). While blocks around the active site residues are conserved throughout all sequences, the residual sequences of CE1 family members do not align with Fll4_CE1a or Fll4_CE1b. Even though sequences of AXEs (e.g., 5X6S) and FAEs (e.g., 8IY8) are represented among the CE1 members, no similarity to the Fll4 domains could be observed (Figure S8b, Supporting Information). Structural comparison of Fll4_CE1a and Fll4_CE1b with CE1 members (Figure S9a,c, Supporting Information) also shows negligible similarity (RMSD > 6). The homologous sequences (Figure S8a,b, Supporting Information, green box) identified through BLAST exhibit significant similarities, extending beyond the active site residues (33–49% sequence identity). Some of these homologs were previously described as putative members of the CE1 family.^[^ ^35^, ^36^, ^37^ ^]^ However, they are now grouped into the nonclassified CEs^[^ ^22^ ^]^ and are therefore likely to belong to new, so far undescribed CE families. It is likely that the Fll4_CE1a and Fll4_CE1b do not belong to the CE1 family, even though annotations by several tools suggest so, but rather they are members of these undescribed new CE families.

The highest similarity to the Fll4 domains can be found with the enzymes from *D. mossii*. These enzymes show high similarity to the domain architecture of Fll4, even though the domains are not fused in one protein, but in three distinct esterases located in the same PUL.^[^ ^38^ ^]^ The esterases *Dm*CE1A and *Dm*CE1B both feature a CBM48 domain, similar to the central domain (CD) of Fll4, which is as well annotated as a CBM48 using dcCAN_sub (Figure S1, Supporting Information). *Dm*CE1A features a putative CE1 domain at the C‐terminus, while *Dm*CE1B features two putative CE1 domains located at both sites of the CBM48 domain. This domain architecture is therefore similar to the one found in Fll4. Additionally, a third esterase is located in the PUL, putative *Dm*CE6A. Therefore, all domains of Fll4 can be found distributed across the *D. mossii* PUL. As the PUL from *D. mossii* encodes several xylan‐targeting enzymes like GH10 and GH43,^[^ ^38^ ^]^ it is likely that they target feruloylated and acetylated xylans just like enzymes from PULII from *Flavimarina* sp. Hel_I_48. Fll4_CE1a shows strong similarity (71.09% percent identity) to *Dm*CE1B (Figure S8a,b, Supporting Information). To distinguish between the N‐terminal (nt) and C‐terminal (ct) CE1 domain of the protein, both sequences were used for the alignment. It is clearly evident that Fll4_CE1a aligns well with *Dm*CE1B_nt, while Fll4_CE1b shows slightly more similarity to *Dm*CE1B_ct than to *Dm*CE1B_nt. The same applies to the structural alignment of AlphaFold models (except *Dm*CE1B_ct, 7BV5) of all proteins (Figure S9b,c, Supporting Information). *Dm*CE1B_nt and Fll4_CE1b show a very strong similarity with an RMSD of 0.295. As mentioned for the Fll4 homologues deriving from the PDB, the esterases of *D. mossii* were described as potential members of the CE1 family. However, as previously reported, it is more likely that Fll4_CE1a, Fll4_CE1b, *Dm*CE1A and *Dm*CE1B all belong to the same, so far not classified CE family.

For Fll4_CE6, a comparison with characterized CE6 members was performed accordingly, revealing a very high similarity to all of them (Figure S10a,b, Supporting Information). Among those also *Dm*CE6A shows a high sequence (68.40%) and structural similarity (RMSD 0.261).

### 2.2 Functional Investigation of the Multimodular CEs

Through investigation of the single domains of Fll4, a precise insight of the different activities mediated by the domains and potential synergistic effects should be addressed. Moreover, the Fll1_CE3 domain was further investigated to determine its natural activity, as it was not previously observed to be active toward several esterase substrates.^[^ ^11^ ^]^ After testing different expression media all enzymes could be obtained as soluble proteins from *Escherichia coli* and were further purified via an N‐terminal His‐Tag using gravity flow‐immobilized metal affinity chromatography (Figure S11, Supporting Information). For the full length Fll4, which has about 100 kDa, impurities could not be avoided during purification.

Moreover, during some expressions, prominent bands around 60 and 35 kDa may also indicate partial fragmentation of the full‐length enzyme. Unfortunately, these fragments could not be completely separated from the full‐length enzyme for further experiments.

**Figure 2:**
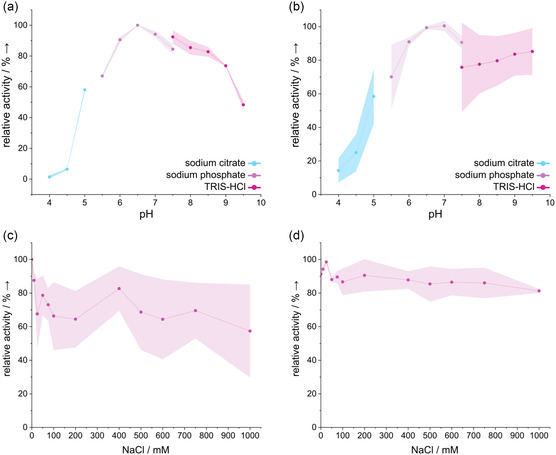
Biochemical characterization of Fll1_CE3 and Fll4 using *p*NP‐acetate. Shown are the pH profile and salt tolerance of a,c) Fll1 and b,d) Fll4. Activity was determined using final concentrations of 1 mm *p*NP‐acetate, 50 mm buffer, 100 mm NaCl, 5% DMSO, and 25 μg mL of the respective enzyme. For NaCl screening, 50 mm TRIS‐HCl pH 8.0 was used with the respective amounts of NaCl. For all conditions, a negative control without enzyme was used and subtracted from the values of the enzyme reactions. The highest activities were set as 100% and relative activities were calculated. Mean values and standard deviations were calculated from technical triplicates. Standard deviations are shown as colored shadows.

Initial screenings of these enzymes toward *p*NP‐acetate were performed to characterize the pH profile and their halotolerance using NaCl (**Figure** **2**). As Fl6 was only slightly active on *p*NP‐acetate, no conclusive results could be obtained for the performed assays. Fll1_CE3 showed a preference toward neutral pH values with an optimum at pH 6.5 but also remained most activity up to a pH value of 9.0. Similar preferences were observed for Fll4 with an optimum at pH 7 and even higher stability at pH values up to 9.5. Also, both enzymes retained most activity in the presence of NaCl up to 1 m, which is to be expected given their marine origin. Other studies have shown that CEs and other CAZymes from marine origin tend to tolerate high salt concentrations during reaction and are likely to show enhanced thermostability at higher salt concentrations.^[^ ^39^, ^40^ ^]^

**Figure 3:**
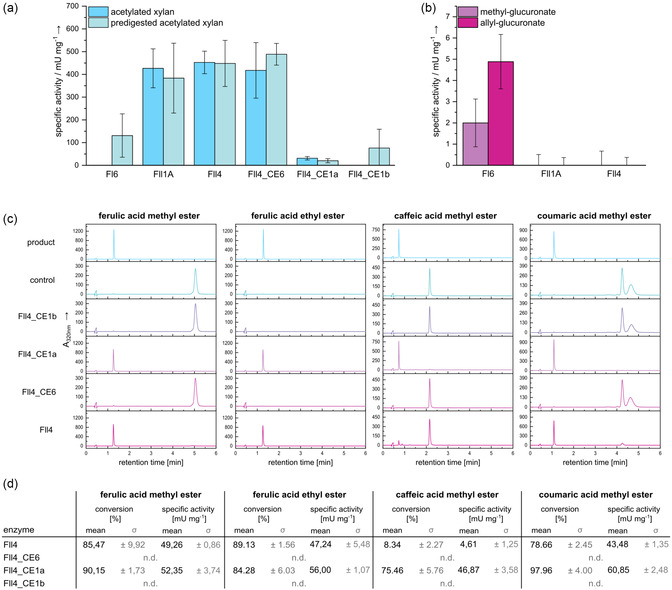
Xylanolytic activities of all full‐length CEs and Fll4 single domains. AXE activity a) of the enzymes toward acetylated xylan and acetylated xylooligosaccharides generated upon prior degradation with an endo‐xylanase. GE activity b) of the enzymes toward methyl glucuronate and allyl glucuronate. c) HPLC chromatograms, conversions, and specific activities of Fll4 and its corresponding single d) toward 1 mm phenolic acid esters for ≈16 h at room temperature shows that Fll4_CE1a mediates FAE activity of full‐length Fll4. Standard deviations are shown in grey. No product formation could be observed for Fl6, Fll1, as well as Fll4_CE6 and Fll4_CE1b (not detectable: n.d.). It has to be noted that the specific activities may be underestimated, as it cannot be assumed that product formation progressed linearly over the entire reaction period. For all assays, a negative control without enzyme was measured and subtracted from the values of the enzyme reactions. Phenolic acid ester conversion was calculated from product peak areas using standard curves of all products (Figure S13, Supporting Information). Mean values and standard deviations were calculated from technical triplicates.

Investigation of the AXE (**Figure** **3**a), the GE (Figure 3b), and the FAE activity (Figure 3c) shows that both Fll4 and Fll1 are active on acetylated xylan polysaccharide, as well as on acetylated xylooligosaccharides that were obtained using a xylanase for prior degradation of the acetylated xylan (Figure 3a). As Fll1 had no natural esterase activity described before, it could be assumed that the expression of the enzyme did not result in an active folding of the esterase domain, resulting in negative results in the AXE assay obtained previously.^[^ ^11^ ^]^ This is also verified by the fact that Fll1_CE3 is indeed active on *p*NP‐acetate as well, which was not observed before for Fll1. As Fll1 did not show any activity toward GE and FAE substrates, it can be assumed that the Fll1_CE3 is solely acting as an AXE, matching its assignment to the CE3 family. The GE function of Fl6 was confirmed, with a higher activity toward the bulkier allyl glucuronate substrate (Figure 3b). Another study of a CE15 enzyme from *Ruminococcus flavefaciens* has shown that enzymes within the CE15 family can exhibit activity toward various acetylated substrates, indicating that the CE15 family may encompass further activities beyond just a GE function.^[^ ^40^ ^]^ Moreover, peptide pattern recognition identified several distinct groups of undescribed CE15 enzymes, which could have other esterase activities.^[^ ^41^ ^]^

Activity assays using the single domains of Fll4 show that the CE6 domain is mediating the AXE activity of the full‐length enzyme (Figure 3a) with a comparable activity for both constructs. The two putative CE1 domains showed negligible deacetylase activity. High performance liquid chromatography (HPLC) analyses using different phenolic acid esters showed product peak formation upon incubation of the esters with Fll4 (Figure 3c). No product formation was observed for Fll1 and Fl6 (not shown). The CE6 and CE1b domain of Fll4 showed no product formation as well, while the activity of the Fll4_CE1a alone is comparable or even higher than the ones of the full‐length Fll4 (Figure 3c), indicating that the CE1a domain is mediating the FAE activity of Fll4. The conversion rates of Fll4_CE1a alone are significantly higher for caffeic and coumaric acid methyl ester (Figure 3d), than for the full‐length enzyme, indicating a loss of activity for the domain when expressed with the other CE domains. However, it must be considered that the purity of the full‐length Fll4 is lower than that of the single domains, which could distort the exact conversions. In comparison to the previously mentioned highly similar proteins from *D. mossi (Dm)i* ^[^ ^38^ ^]^ the observed activity of the Fll4 domains matches their homologues. While *Dm*CE6A is reported to act as a deacetylase on various substrates, *Dm*CE1B and *Dm*CE1B_nt (similar to CE1a) show mainly FAE activity with negligible activity as a deacetylase. Just as for Fll4 and Fll4_CE1a, the activities were overall highly similar, despite truncation of the enzyme. *Dm*CE1A and *Dm*CE1B_ct are both similar to CE1b with CD. While *Dm*CE1A showed minimal FAE activity and no AXE activity, the *Dm*CE1B_ct conversely showed no FAE activity and negligible AXE activity. As the authors emphasized, the lack of activity could indeed be attributed to an insufficient similarity between the model substrates and the natural substrates of the enzymes.^[^ ^38^ ^]^ Therefore, the development of improved model substrates would be of great interest for identifying the natural activity of Fll4_CE1b and *Dm*CE1B_ct.

As the CE1b domain of FII4 did not show activity toward any of the tested substrates, the relevance of the other domains together with the CE1b domain was investigated. As the CE1b did not show activity toward *p*NP‐acetate, it was assumed that the domain is maybe not expressed properly when detached from the rest of the enzyme. Expression of the CE1a and CE1b domain together led to the same specific activity toward *p*NP‐acetate than CE1a alone. Also, the removal of a C‐terminal domain did not lead to any activity observed for CE1b (Figure S12, Supporting Information). This could indicate that the CE1b domain was either not actively expressed or properly folded or it lacks activity against the tested substrates.

Given that polysaccharides, particularly in marine environments, display complex structures, the CE1b domain may possess high specificity toward a substrate that remains unknown for now. Nevertheless, Fll4 exhibited two distinct activities and can therefore be considered a bifunctional AXE/FAE. This bifunctionality is known for terrestrial enzymes where they mainly play a role in the de‐esterification of hardwoods. There are enzymes partially similar to the Fll4, which are comprised of at least two domains, a N‐terminal CE6 domain and a C‐terminal CE1 domain, originating from soil and gut *Bacteroidota*.^[^ ^42^ ^]^

## 3 Conclusion

This study provides insights into the functional and structural characteristics of the three multimodular xylanolytic esterases Fl6, Fll1, and Fll4, originating from two distinct PULs of *Flavimarina* sp. Hel_I_48. The two enzymes Fll1 and Fll4 are encoded in one PUL which is putatively addressing arabinoxylans, and they both have deacetylase activity, implying that multiple AXEs may be necessary for the complete degradation of the substrates targeted by this PUL. As their substrate specificity is hard to investigate due to a lack of commercially available substrates harboring different acetylation patterns, it can only be assumed that those enzymes could potentially address different acetylations in the respective substrates. The comparison of Fll1_CE3 with members of the CE3 family showed no similarity of Fll1_CE3 to this family, even though the annotations suggested this. In contrast high similarity to two unclassified AXEs, 7TOG and 7TOJ^[^ ^33^ ^]^ was found, suggesting that Fll1_CE3 might be a member of a novel CE family.

Fll4 is exhibiting AXE/FAE bifunctionality, which we were able to attribute to the CE6 domain, mediating the AXE function, and the CE1a domain, mediating the FAE function. The third domain showed no activity toward all studied substrates and this needs to be further investigated to unravel the complete function of the three CE domain enzyme Fll4. The middle domain that was assigned as a CBM48 according to dbCAN_sub could play a role in substrate binding as well, as it is reported that for CE1 enzymes harboring a CBM48 domain, substrate recognition is disturbed upon deletion of the binding domain.^[^ ^36^ ^]^ Nevertheless, as the CE1b domain was expressed while still containing this domain, other synergistic effects with the residual domains may have occurred. Moreover, the comparison of the putative CE1 domains of Fll4 reveals high similarity to nonclassified CEs again, suggesting an affiliation of these domains to a so‐far undescribed CE class, rather than the CE1 family.

Alongside many other intriguing enzymes already discovered in marine environments, our findings highlight the immense potential inherent in enzymes from marine organisms. Owing to their often high selectivity or tolerance under diverse reaction conditions, such enzymes can serve as valuable biocatalysts.^[^ ^43^, ^44^, ^45^, ^46^, ^47^ ^]^

Multidomain CAZymes have been extensively studied for decades and are well documented for their roles in polysaccharide degradation, structural diversity, and catalytic efficiency.^[^ ^47^, ^48^, ^49^ ^]^ In the context of marine polysaccharides, their specific functions, particularly in relation to the removal of modifications such as ester‐linked compounds, side chains, or sulfation, remain an area of ongoing investigation. These modifications are known to influence the activity of GHs, and their removal may enhance performance of endo‐acting enzymes.^[^ ^46^, ^50^ ^]^ Therefore, a deeper understanding of naturally occurring multidomain enzymes is essential to uncover their full potential, optimize their applications, and to explore their advantages over conventional single‐enzyme cascades.

## 4 Experimental Section

4.1

4.1.1

##### Annotation

The domain architecture of the studied enzymes was predicted using hmmscan (v3.3.2) against the dbCAN‐HMMdb‐V12,^[^ ^51^ ^]^ the dbCAN_sub, and the PFAM^[^ ^52^ ^]^ (r35.0) databases. The results were then filtered using the hmmscan‐parser.sh script provided by dbCAN with an e‐value cut‐off ≤1e‐5 and a minimum coverage of 30%.

##### Protein Structure Prediction and Visualization

Prediction of all enzyme structures was carried out using the AlphaFold3 web server.^[^ ^25^ ^]^ Editing and visualization of the structures was done using PyMol software. For electrostatics prediction of the AlphaFold3 models of all enzymes, PQR2PDB web tool (https://server.poissonboltzmann.org/pdb2pqr, Amber Forcefield) was used for preparation of the structures. Afterwards APBS Electrostatics Plugin for PyMol was used (default settings) for electrostatic map calculation and molecular surface visualization. Conserved residues were visualized using ConSurf web tool (https://consurf.tau.ac.il).^[^ ^27^ ^]^ As the CE3 domain of Fll1 shows insufficient data for determination of conservation scores when calculating the full‐length protein, the conservation score of the domain was additionally calculated using the single domain as an input.

##### Gene Cloning of Single Domains

The pET28a(+) plasmids carrying the genes corresponding to the amino acid sequences listed in Table S1, Supporting Information were generated as previously described.^[^ ^11^ ^]^ Single‐esterase domains of Fll4 were cloned via deletion of the remaining domains using NEBaseChanger for primer design (Table S2, Supporting Information). For PCR Q5‐Polymerase was used following the recommended protocol of the manufacturer. The PCR‐product was used for the KLD reaction, again following the manufacturers recommendations, and chemically transformed into *E. coli* TOP10 cells. The success of the deletions was verified by sequencing of the isolated plasmids before further use.

##### Protein Production and Purification

This was performed in *E. coli* Bl21 (DE3) using pET28a(+) plasmids carrying the corresponding genes. Overnight cultures were used to set the expression culture to an OD_600nm_ of 0.1. After incubation at 37 °C until the OD_600nm_ reached 0.6–0.8, the culture was cooled to 20 °C and 0.5 mm IPTG was added. After 20 h the cells were harvested by centrifugation at 4,500 × g and 4 °C.

Fl6 and Fl6_CBM were produced in LB media (Miller) with 50 μg mL^−1^ kanamycin in baffled flasks at 140 rpm for 20 h. Fll1_CE3 was expressed in TB media with 50 μg mL^−1^ kanamycin in baffled flasks at 140 rpm for 20 h. Full‐length Fll1 could not be expressed as soluble. Fll4 and its domains were expressed in TB media with 50 μg mL^−1^ kanamycin in unbaffled flasks at 180 rpm for 20 h.

Lysis was carried out using ultrasonication on ice (2 × 3 min, 50% power, 50% cycle time) followed by centrifugation at 10,000 × g for 10 min at 4 °C. The resulting supernatant was applied to ROTIGarose‐His/Ni Beads (Roth) in a column with a volume (Cv) of 4 mL, equilibrated with lysis buffer (50 mm TRIS‐HCl, pH 8.0, 300 mm NaCl). The column was washed with 20 Cv of 20 mm imidazole in 50 mm TRIS‐HCl, pH 8.0, 300 mm NaCl, and the enzyme was eluted using 2 Cv of 300 mm imidazole in 50 mm TRIS‐HCl, pH 8.0, 300 mm NaCl. Imidazole was removed using PD‐10 columns (GE Healthcare) equilibrated with 50 mm TRIS‐HCl, pH 8.0, 25 mm NaCl. The enzyme was stored at –20 °C, and to prevent activity alterations, thaw‐freeze cycles were avoided. Protein concentration was determined using Pierce BCA Protein Assay Kit (Thermo Scientific).

##### Determination of Protein Melting Temperatures Using Nano‐DSF

For monitoring putative binding events of Fl6_CBM with several xylose containing polysaccharides, melting temperatures of the protein were measured upon absence and presence of the putative substrates using nano DSF (Nano‐DSF). Mixtures were prepared using final concentrations of 150 μg mL^−1^ protein, 5 mg mL^−1^ substrate, and 50 mm TRIS‐HCl buffer pH 8.0 with 100 mm NaCl. The unfolding of the protein was observed from 20 to 90 °C with a 1 °C min^−1^ gradient.

##### Acetate Release Assay

Acetylated birchwood xylan (AcX, Megazyme Ltd.) was enzymatically pretreated overnight with a purified GH10 β‐1,4‐xylanase (UniProt A0A1D7XPY9).^[^ ^53^ ^]^ After treatment, the xylanase and remaining polysaccharides were removed with a 10 kDa cutoff filter, resulting in purified acetylated oligosaccharides (predigested acetylated xylan). The esterases (30 μg mL^−1^) were incubated with substrates in 50 mm TRIS‐HCl buffer (pH 8.0) containing 100 mm NaCl at room temperature for 16 h. Released acetic acid was measured using an acetic acid kit from R‐Biopharm (Darmstadt, Germany) modified for microtiter plate format and reduced to 10% of the original protocol. Substrate‐only controls were included and their values were subtracted from the test results to calculate the acetic acid concentrations. All experiments were performed in triplicate, and mean values with standard deviations were reported.

##### Glucuronic Acid Assay

GE activity was evaluated using the K‐URONIC assay (Megazyme Ltd) with methyl d‐glucuronate (BioSynth) and allyl d‐glucuronate (BioSynth) as substrates. The reactions were prepared with 100 μg mL^−1^ enzyme and 2 mm substrate in 50 mm sodium phosphate buffer (pH 6) and incubated according to the assay protocol. After 30 min, the reaction was stopped by cooling to 4 °C. A detection solution (50 μL) was added to the reaction mixtures (200 μL) in microtiter plates, and absorbance at 340 nm was measured every 30 s over 60 min. A glucuronic acid standard was used to verify the endpoint of the detection reaction. Absorbance changes were calculated after subtracting values obtained from enzyme‐free controls. All experiments were performed in triplicate, and mean values with standard deviations were reported.

##### Phenolic Acid Assay

FAE activity was studied using different phenolic acid esters as substrates. Enzyme (100 μg mL^−1^) was incubated with 0.4 mm phenolic acid esters in 100 mm TRIS‐HCl (pH 8.0) containing 100 mm NaCl. Reactions were carried out at room temperature for 16 h with shaking at 1,000 rpm. The reaction was stopped by heating the mixture to 80 °C for 10 min. To extract the product, 50 μL of methanol was added, vortexed for 30 s, and centrifuged at 13,000 × g for 5 min. The supernatant (5 μL) was analyzed via ultra high performance liquid chromatography using a Kinetex 2.6 μm C18 100 Å column and a mobile phase of 80% water and 20% acetonitrile with 0.1% formic acid. Absorbance at 320 nm was used to quantify phenolic acid formation based on a standard curve. All experiments were performed in triplicate, and mean values with standard deviations were reported.

##### *p*NP‐Acetate Assay

Esterase activity was determined using final concentrations of 1 mm *p*NP‐acetate, 50 mm TRIS‐HCl pH 8.0 (if not stated otherwise), 100 mm NaCl, 5% DMSO, and ≈25 μg mL^−1^ of the respective enzyme. The product formation was monitored continuously for 15 min every 30 s, measuring the absorption at 348 nm^[^ ^54^ ^]^ at room temperature. The slope in the linear area of the assay was used for calculation of specific activities (*ε* = 15,000 M^−1^ cm^−1^). pH profiles were determined using 50 mm of the respected buffers instead of TRIS‐HCl. NaCl profile was determined using 50 mm TRIS‐HCl pH 8.0 with respective amounts of NaCl. All experiments were performed in triplicate, and mean values with standard deviations were reported.

##### Sequence Alignments

Protein sequences of the full length enzymes were used for the identification of homologous proteins from the PDB^[^ ^55^ ^]^ using NCBI BLAST.^[^ ^56^ ^]^ The N‐terminal His‐Tag and the thrombin cleavage site of the protein sequences were removed prior to analyses. Different homologs were selected for multiple sequence alignment using T‐coffee,^[^ ^57^ ^]^ default settings. Residues were colored using Clustal, conservation interval was set to 10. For structural comparison the homologous structures were retrieved from PDB and aligned using the PyMol alignment tool.

## Conflict of Interest

The authors declare no conflict of interest.

## Supporting information

Supplementary Material
