# Supplementary material for: Biochemical Characterization of Multimodular Xylanolytic Carbohydrate Esterases from the Marine Bacterium Flavimarina sp. Hel_I_48
Source: Chembiochem. 2025 Apr 8;26(13):e202500058. doi: 10.1002/cbic.202500058 (PMC12247016; doi:10.1002/cbic.202500058)
Supplement: Supplementary file 1 — Supplementary Material [file CBIC-26-e202500058-s001.zip › cbic.202500058-sup-0001-suppdata-S1.pdf]

Supporting Tables

Table S1: Amino acid sequences of each expressed construct. All sequences contain an N-terminal 6x His-Tag and a thrombin cleavage site for purification (underscored). All constructs were expressed using pET28a(+) vector and *E. coli* BL21 (DE3) as expression hosts.

**Construct**	**Expressed amino acid sequence (N → C)**	**kDa**
FI6	MGSSHHHHHHSSGLVPRGSHMQLPLVYNSENTGSNCNMPPLPDPGDLQNIPKLPDPFAWSNGIGRSTQFSDWECRRNEIKAEIQEYEIGLKP<br>IAPADITASYADGVLTVNVMKDGETLTLTSQVTMPEGDGPFPVVIGMNSATGSLPDSLFEGVIKIPFMHNQVVSYSQGSRNPDDPYFKLYPEFH<br>PDEDTYLMGNYSAWSWGVSRLIDGIAMVQDQLRADVSHIAVTGCSYAGKMALFAGAFDERIALTIAQESGGGGAPAWRVSETIGNVEKIDNTN<br>YSWFLPNMGAKFDGRVGILPHDHHELMAMVAPRALLITGNTSYEWLANPSAYVSARATEEVYKALGIEDRFGFYIDGDHNHCAIPESEQPSIRA<br>FVDKFLFDDTSAETEIRVNPYGDTVDYRSWISGWTDTDPNTPNIMINSPEDNATFEAPATVTITATVSDENDDVQKVEFFNGAELLGEDADAPY<br>AFTWENLEGGTYQLSAKATDAQGLLGYSNVIKITVTTPPSSVYKVTTPPEIDGMIDDLWENAKINVLEAENVLLGDSIEETDLSGIAKVIWDDTFM<br>YILAEVTDDIKLNDSPNAIYQDDNIEIYLDGNNGKTNTYEPGNDVQYTFRWDDGDFVGTSNGISTEGIAYVMQGTDTGYIFEAKIPLANLGITTED<br>GMMIGFDFMINDDDDGGDRDAKLSWHATNDSAYQNTSVFGTVVLAGETL	77.62
FI6_CBM	MGSSHHHHHHSSGLVPRGSHMIDGMIDDLWENAKINVLEAENVLLGDSIEETDLSGIAKVIWDDTFMYILAEVTDDIKLNDSPNAIYQDDNIEIYL<br>DGNNGKTNTYEPGNDVQYTFRWDDGDFVGTSNGISTEGIAYVMQGTDTGYIFEAKIPLANLGITTEDGMMIGFDFMINDDDDGGDRDAKLSW<br>HATNDSAYQNTSVFGTVVLAGETL	23.32
FII1_CE3	MGSSHHHHHHSSGLVPRGSHMCSSTQDSVETTELKNWVGTWSTAPQLVEPHNMPPEPGLTGNTLRQVLEVSIGGKQLRMKFSNEFSANPL<br>EIHSVQIAVSEDSSAIAVNTSRMLMFEGQEAVTIPAGKAIFSDPIKFDLNPRMQLAVTIAYGQTPADVTGHPGSRTTSYLLSGHQISPEANFSKAI<br>KTDHWYTINSIEVKSNETTAAVAIIGNSITDGRGSGTNKQNRWPDILSQQLLKNQDTENIGVLNMGIGGNAVLQGGLGPTALDRFDRDILQQNGI<br>KWLIIMEGVNDLGATPDSAAAFKVADGLIAAYEEMIEKAHKKNIKVLGGTITPIKESFYYKEYREQARQKINDWIRTSGKFDGVVDFDKVIRNPEN<br>TAVIRENAHDGDHLHPNEYGYEL	43.85
FII1_GH43	MGSSHHHHHHSSGLVPRGSHMLLHITASDGYDLWLRYVPIENQTVLTDYQDIFRSVYFEGDSDKHLVSKDELKRGTQGMLGQEIELLPSYSDK<br>NTLVIAPATNLNEVLSAGLKKDLKTIGKEGFIIKTVQNKGKNILVVTANTSTGILYGTFRFLMLMQQNQDFSAINQVEKPKVDLRILNHWDNLDRT<br>VERGYAGFSIWNWQKLPGFIDQRYIDYARVNASIGINGTVLTNVNANALVLTPQYIEKVAALADVFRPYGIKVYLTARFSAPIEIGGLETADPLDP<br>KAKQWWKDKADEIYKKIPDFGGFLVKANSEGQPGPQNYGRNHVDGANMLAEAVAPHNGIVMWRAFVYSEDDATDRAKQAFSEFVPMDGKF<br>LDNVIIQVKNGPIDFQPREPFHPMFGAMPKTPLMIEFQNTQEYLGFSTHLAYLPKLYEEVLQADTYQKGKGSTVAKVIDGSLHDKKLTGMAGVA<br>NIGTDINWTGHPFAQANWYGFGRLAWDPELDSRVIAKEWLRATFSNNEEFVDTMAKVMIDSREAVVNYMTPLGLHHMMATGHHYGPGPWV<br>DNLSRPDWNPVYYHKADSLGIGFDRTKSGSDAVDQYAPELAKKFNDPRTTPEELLLWFHHLPWEYTLESGKSLWDGIALKYQKGVDEVKTMA<br>NTWKKMQPYVNKEQFEEVRMLLEIQLKEAKWWRDACLLYFQQFSQKPLPEGVEKPAESLEYYEALRFPFAPGIRPQWD	82.87
FII4	MGSSHHHHHHSSGLVPRGSHMQDPDFHIYLAFGQSNMEGAAKIEPQDTMNLNERFKVLEAVDCPELGREKGKWYTAKAPLCRCKTGLTPTD<br>YFGREMIKNLPENVKVGVINVAVGGCKIELFDQDNFQSYVETAPEWLQNMVKEYDGNPYARLVEMAKIAQKDGVIKGILMHQGESNTGDEEW<br>PNKVKGVYDDLIKDLNLDPKKVPLLAGEMVSEEQGGACASMNEIIARLPGVIPNAHVISSKGCTAVSDHLHFTSEGYRKLGRRYAQQMLQLQG<br>VEILERQAPEGFDTEQEDIPHGKIDSISYKSKTVGTTRKALVYTPPKYSKTKKYPVLYLLHGIGGDEKEWLRGGKPQVILDNLHAEGKIEPMIVVM<br>PNGRAMKDDRAVGNIFDSTKVEAFANFEGDLLNDLVPFVEKNYPVMKNRENRAIAGLSMGGGQTLNFGLGNLDTFSWVGAFSAAPNTKTPQE<br>LVPSPEKAKNSLNLLWISCGDEDGLLPYSQRTHEYLAKNKVPHIYYVEPGVHDFKVWKNGLYMFSKMLFKPVDKTVFNDYSILGSTVATNVGK<br>SKFPQILPNGKAMFQLKAPDASDVKLDLGRKYEMEHDGDGMWRVTTDSISEGFHYYSIIVDGVAIADPNSETFYGMGRMASGIEVPFKGDDYY<br>ALKDVPHGDIIMEQYFSPVLNSWRKFFVYTPPGYEKNTDEKYPALYIYHGGGEDERGWAQQGKTNLILDNLIAEGKAKPMLVIMPDGNMPVSA<br>FDENGLKMFENELIKGIIPQVEKEYRVIKNPKSRALAGLSMGGIQTLYAGVQNTDLFSSLGVFSSGWIGKDNEIADGQYEFMTKNSAKINKNLDN<br>FWISMGGERDIAYENGKRMISRFDDMGIRYTYSEYPGGHAWPVWRHDLYKFAPLLFTN	100.34
FII4_CE6	MGSSHHHHHHSSGLVPRGSHMQDPDFHIYLAFGQSNMEGAAKIEPQDTMNLNERFKVLEAVDCPELGREKGKWYTAKAPLCRCKTGLTPTD<br>YFGREMIKNLPENVKVGVINVAVGGCKIELFDQDNFQSYVETAPEWLQNMVKEYDGNPYARLVEMAKIAQKDGVIKGILMHQGESNTGDEEW<br>PNKVKGVYDDLIKDLNLDPKKVPLLAGEMVSEEQGGACASMNEIIARLPGVIPNAHVISSKGCTAVSDHLHFTSEGYRKLGRRYAQQMLQLQG<br>V	30.80
FII4_CE1a	MGSSHHHHHHSSGLVPRGSHMLQGVEILERQAPEGFDTEQEDIPHGKIDSISYKSKTVGTTRKALVYTPPKYSKTKKYPVLYLLHGIGGDEKE<br>WLRGGKPQVILDNLHAEGKIEPMIVVMPNGRAMKDDRAVGNIFDSTKVEAFANFEGDLLNDLVPFVEKNYPVMKNRENRAIAGLSMGGGQTL<br>NFGLGNLDTFSWVGAFSAAPNTKTPQELVPSPEKAKNSLNLLWISCGDEDGLLPYSQRTHEYLAKNKVPHIYYVEPGVHDFKVWKNGLYMFS<br>KMLFKPVD	31.83
FII4_CE1b	MGSSHHHHHHSSGLVPRGSHMKTVFNDYSILGSTVATNVGKSKFPQILPNGKAMFQLKAPDASDVKLDLGRKYEMEHDGDGMWRVTTDSIS<br>EGFHYYSIIVDGVAIADPNSETFYGMGRMASGIEVPFKGDDYYALKDVPHGDIIMEQYFSPVLNSWRKFFVYTPPGYEKNTDEKYPALYIYHGG	42.73

	GEDERGWAQQGKTNLILDNLIAEGKAKPMLVIMPDGNMPVSAFDENGLKMFENELIKGIIPQVEKEYRVIKNPKSRALAGLSMGGIQTLYAGVQNTDLFSSLGVFSSGWIGKDNEIADGQYEFMTKNSAKINKNLDNFWISMGGERDIAYENGKRMISRFDDMGIRYTYSEYPGGHAWPVWRHDLYKFAPLLFTN	
Fll4_CE1a_CE1b	MGSSHHHHHHSSGLVPRGSHMLQGVEILERQAPEGFDTEQEDIPHGKIDSISYKSKTVGTTRKALVYTPPKYSKTKKYPVLYLLHGIGGDEKEWLRGGKPQVILDNLHAEGKIEPMIVVMPNGRAMKDDRAVGNIFDSTKVEAFANFEGDLLNDLVPFVEKNYPVMKNRENRAIAGLSMGGGQTLNFGLGNLDTFSWVGAFSAAPNTKTPQELVPSPEKAKNSLNLLWISCGDEDGLLPYSQRTHEYLAKNKVPHIYYVEPGVHDFKVWKNGLYMFSKMLFKPVDKTVFNDYSILGSTVATNVGKSKFPQILPNGKAMFQLKAPDASDVKLDLGRKYEMEHDGDGMWRVTTDSISEGFHYYSIIVDGVAIADPNSETFYGMGRMASGIEVPFKGDDYYALKDVPHGDIIMEQYFSPVLNSWRKFFVYTPPGYEKNTDEKYPALYIYHGGGEDERGWAQQGKTNLILDNLIAEGKAKPMLVIMPDGNMPVSAFDENGLKMFENELIKGIIPQVEKEYRVIKNPKSRALAGLSMGGIQTLYAGVQNTDLFSSLGVFSSGWIGKDNEIADGQYEFMTKNSAKINKNLDNFWISMGGERDIAYENGKRMISRFDDMGIRYTYSEYPGGHAWPVWRHDLYKFAPLLFTN	72.24
Fll4_CE1b_ Δlg	MGSSHHHHHHSSGLVPRGSHMKGDDYYALKDVPHGDIIMEQYFSPVLNSWRKFFVYTPPGYEKNTDEKYPALYIYHGGGEDERGWAQQGKTNLILDNLIAEGKAKPMLVIMPDGNMPVSAFDENGLKMFENELIKGIIPQVEKEYRVIKNPKSRALAGLSMGGIQTLYAGVQNTDLFSSLGVFSSGWIGKDNEIADGQYEFMTKNSAKINKNLDNFWISMGGERDIAYENGKRMISRFDDMGIRYTYSEYPGGHAWPVWRHDLYKFAPLLFTN	31.00

Table S2: Primers for the generation of Fll4 single domains. Primers were designed using NEBaseChanger and PCR was carried out using the manufacturers recommendations. All plasmid sequences used in this study are available in an additional xlsx file.

Primer name	Nucleotide sequence (5' → 3')	Template	Deletion	Product
Fll4_delCE1b_f	TAAGCGGCCGCACTC	pET28a(+)_Fll4	CE1b	pET28a(+)_Fll4_CE6_CE1a
Fll4_ delCE1b_r	ATCAACCGGCTTAAACAGC			
Fll4_CE1b_f	AAAACCGTTTTTAATGATTATAGCAT	pET28a(+)_Fll4	CE6 & CE1a	pET28a(+)_Fll4_CE1b
Fll4_ CE1b _r	CATATGGCTGCCGC			
Fll4_CE6_f	TAAGCGGCCGCACTC	pET28a(+)_Fll4	CE1a & CE1b	pET28a(+)_Fll4_CE6
Fll4_CE6_r	CACACCCTGCAGCTG			
Fll4_CE1a+b_f	CTGCAGGGTGTGGAAATTC	pET28a(+)_Fll4_CE6_CE1a	CE6	pET28a(+)_Fll4_CE1a
Fll4_CE1a+b _r	CATATGGCTGCCGCG			
Fll4B_ΔMD_f	AAAGGTGACGATTATTATGCAC	pET28a(+)_Fll4	CE6, CE1a & MD	pET28a(+)_Fll4_CE1b_ΔMD
Fll4B_ ΔMD_r	CATATGGCTGCCGCG			

Table S3: Growth and yield of expressed full-length Fll4 and its corresponding CE domains. Expression was carried out in 20 mL of TB substituted with 50 ug mL^{-1} kanamycin in 250 mL baffled flaks. Protein expression was induced using 0.5 mM IPTG and took place for 20h at 20 °C. Protein concentration measurements were carried out in duplicates, mean values and standard deviations were calculated.

Enzyme	OD_{600nm}	Protein yield per volume of culture [mg L^{-1}]
FlI4	13,66	345 ± 5,43
FlI4_CE6	13,14	309 ± 10,68
Fll4_CE1a	9,54	68.5 ± 3,85
Fll4_CE1b	17,98	273.5 ± 15,93

Supporting Figures

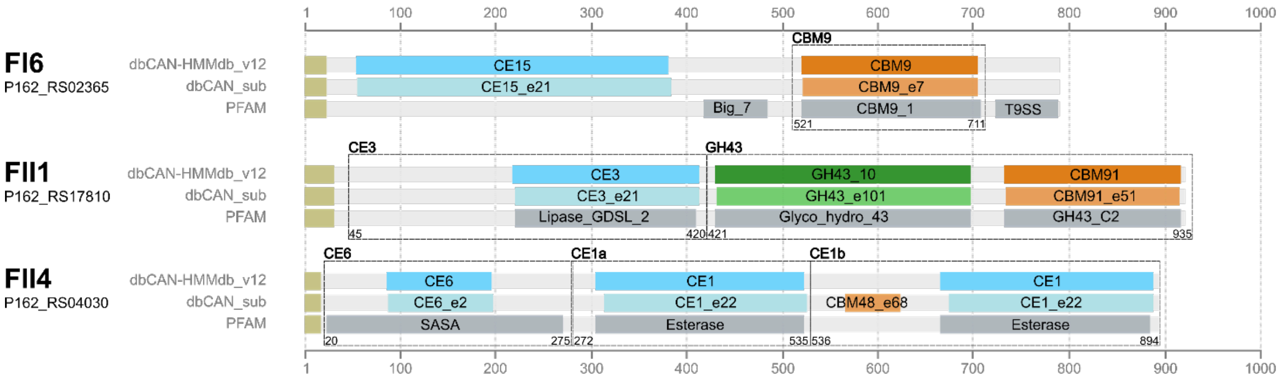

Figure S1: Annotations of the protein sequences of Fl6, Fll1 and Fll4 based on dbCAN-Hmmdb_v12, dbCAN_sub and PFAM. CE domains are shown in cyan, GH domains are shown in green and CBM domains are shown in orange. Dashed line boxes correspond to the part of the protein sequence that is expressed for each domain (named on top of the box) while the numbers in the corners of each box belong to the first and last amino acid expressed in the construct. For example, the CE1a domain of Fll4 is expressed as the construct Fll4_CE1a and contains amino acid 272 to 535 of the full-length protein sequence of Fll4. The gene locus tag of the originating genes in *Flavimarina* sp. Hel_I_48 can be found underneath the enzyme name. The central domain of Fll4 corresponds to the domain annotated as CBM48_e68 by dbCAN_sub.

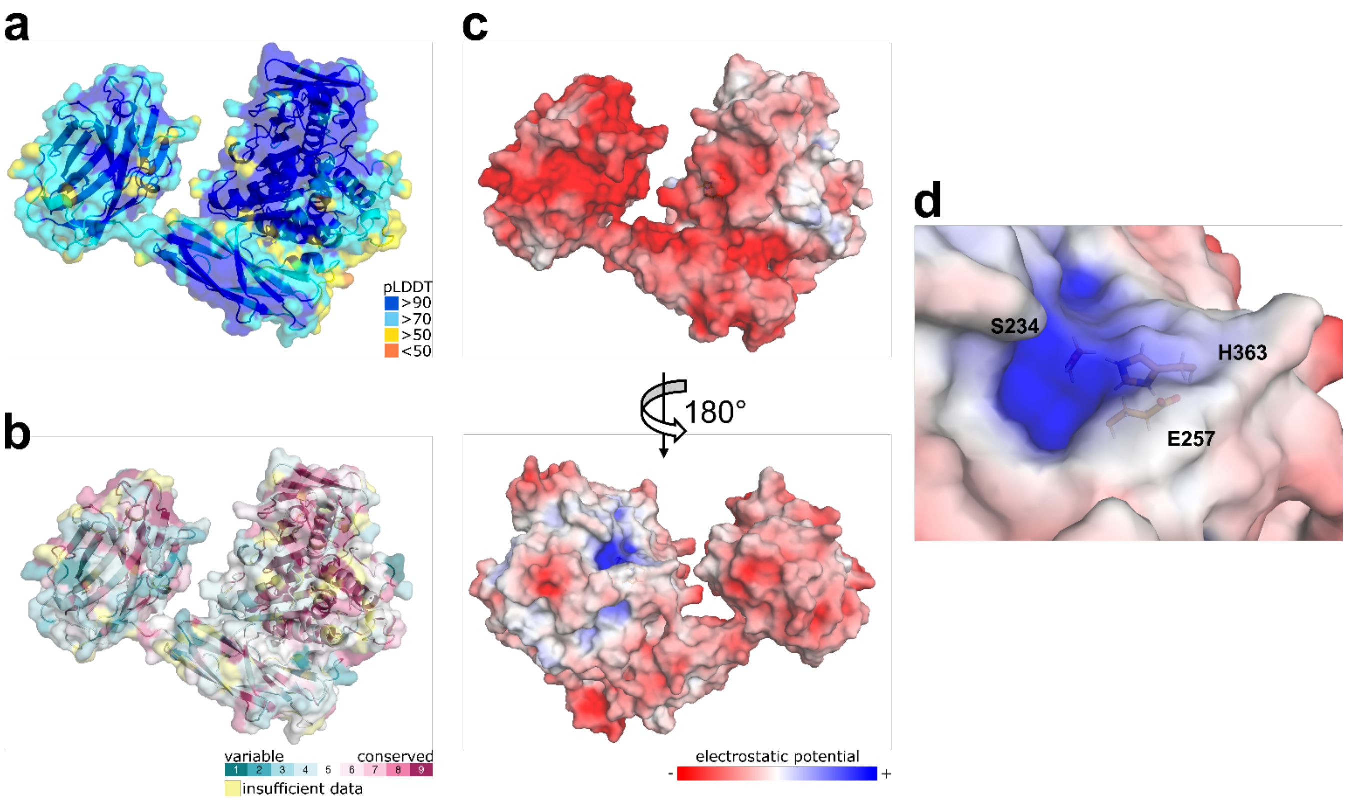

Figure S2: AlphaFold3 model of Fl6. Cartoon and surface visualization of the confidence score (pLDDT) colored model (**a**), conservation score colored model (**b**) and the electrostatic potential coloured model (**c**). The electrostatic potential around the active site (**d**) of the CE3 domain is visualized with semi-transparent surface and active site residue side chains are represented as orange sticks.

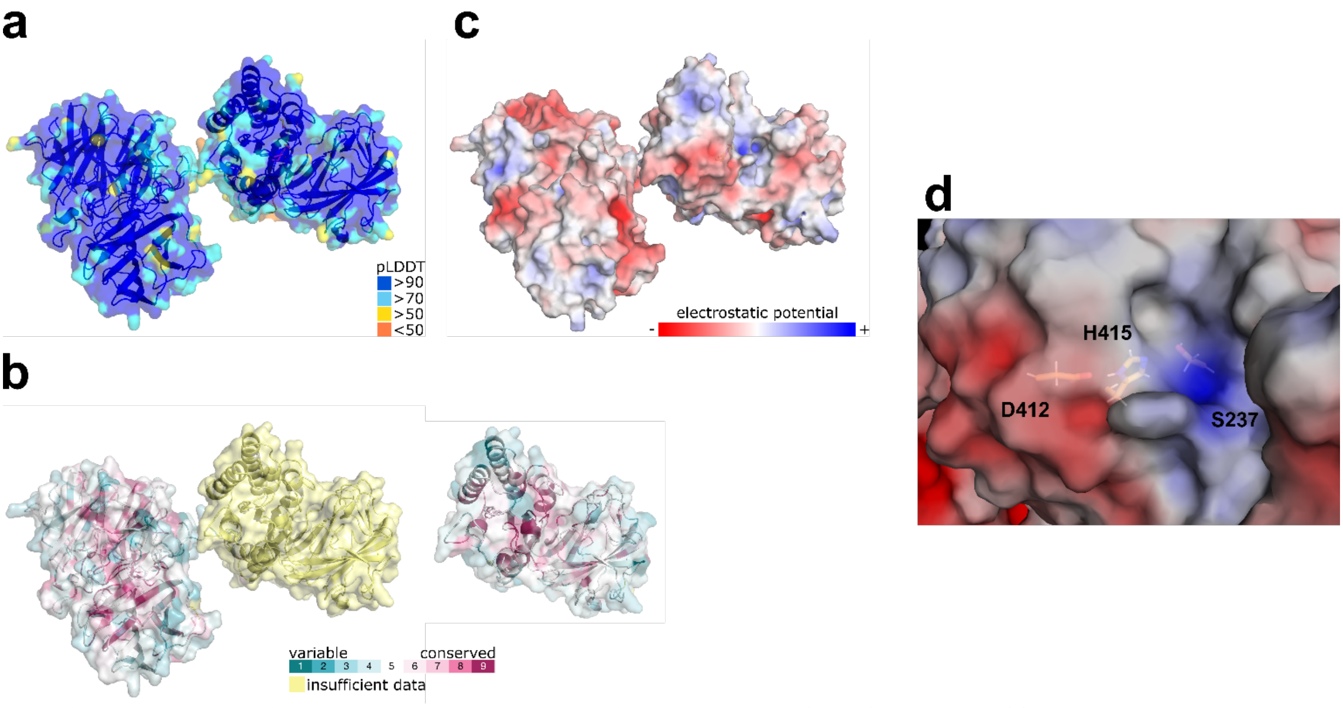

Figure S3: AlphaFold3 model of FII1. Cartoon and surface visualization of the confidence score (pLDDT) colored model (**a**), conservation score colored model of full-length FII1 and FII1_CE3 (**b**) and the electrostatic potential colored model (**c**). The electrostatic potential around the active site (**d**) of the CE3 domain is visualized with semi-transparent surface and active site residue side chains represented as orange sticks.

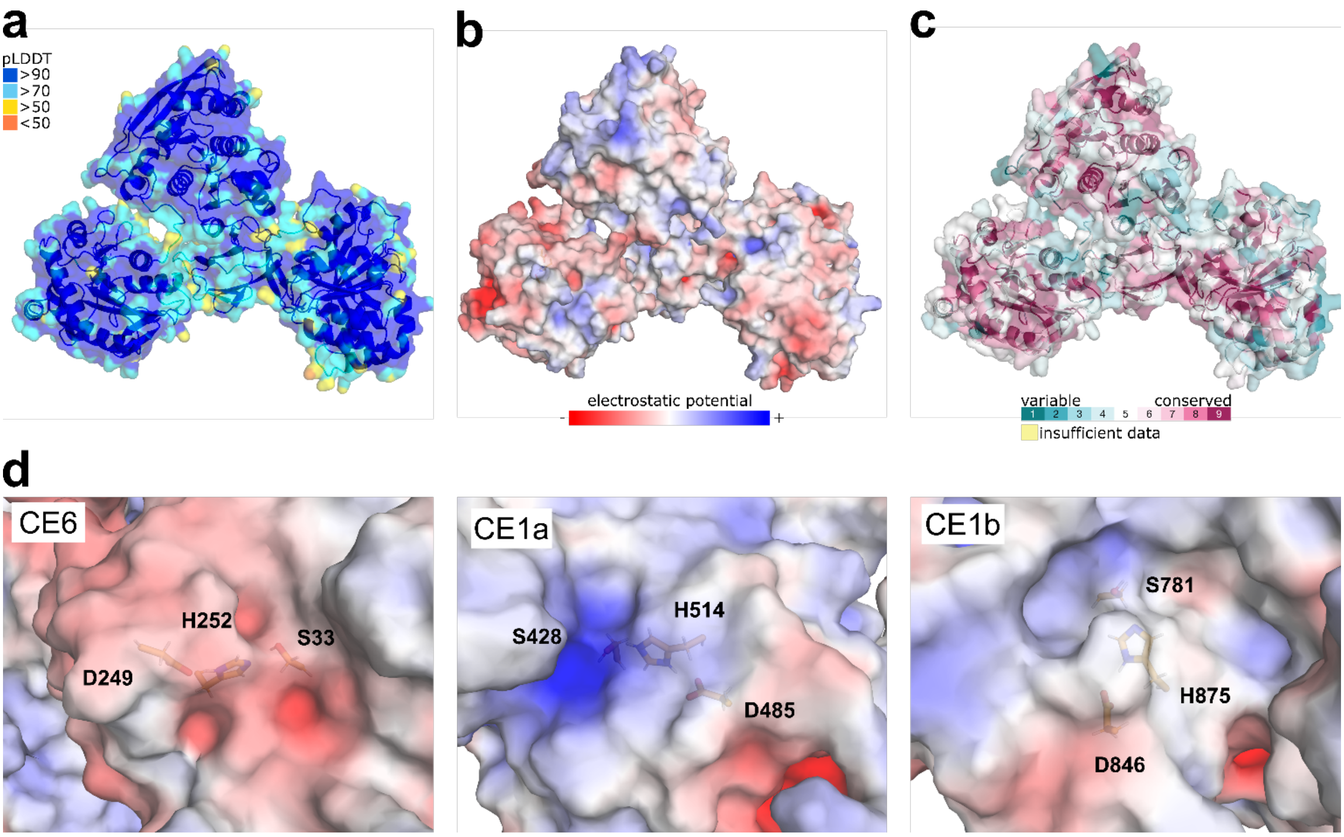

Figure S4: AlphaFold3 model of FII4. Cartoon and surface visualization of the confidence score (pLDDT) colored model (**a**), the electrostatic potential colored model (**b**) and conservation score colored model (**c**). The electrostatic potential around the active sites (**d**) of all esterase domains is visualized with semi-transparent surface and active site residue side chains represented as orange sticks.

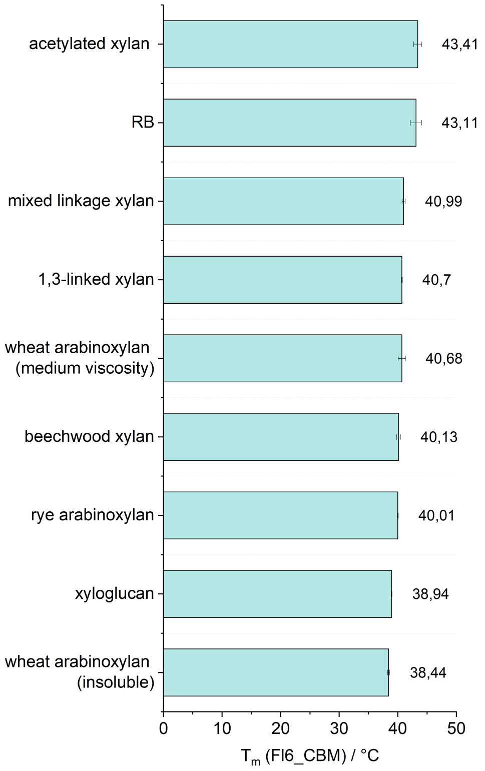

Figure S5. Melting temperatures of Fl6_CBM in reaction buffer (RB) and in the presence of several polysaccharide substrates determined using Nano-DSF. As the melting temperature was not increased upon presence of several polysaccharides no binding event could be observed. All measurements were carried out in final concentrations of 50 mM TRIS-HCl pH 8.0 and 100 mM NaCl with 5 mg mL^{-1} of the corresponding polysaccharide. Mean values and standard deviations were calculated from technical triplicates.

a

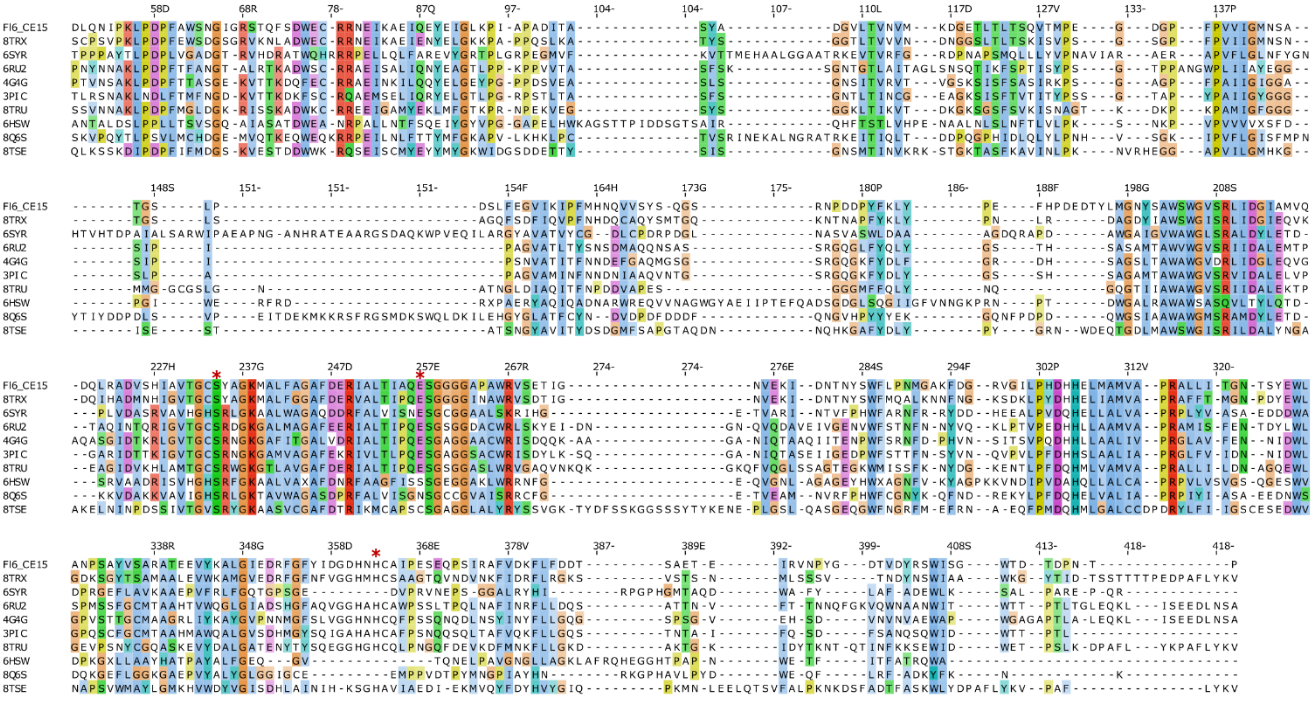

b

	Fl6		Fl6_CE15	
	Query Coverage [%]	Percent Identity [%]	Query Coverage [%]	Percent Identity [%]
**8TRX**	56	54.78	97	55.56
**6RU2**	52	35.96	97	35.96
**3PIC**	51	34.82	91	35.07
**8TRU**	56	33.83	98	34.67
**4G4G**	49	35.36	91	35.36
**8TSE**	49	27.25	89	27.42
**6SYR**	22	36.60	41	36.60
**8Q6S**	25	32.39	47	32.39
**6HSW**	22	32.69	41	32.69

c

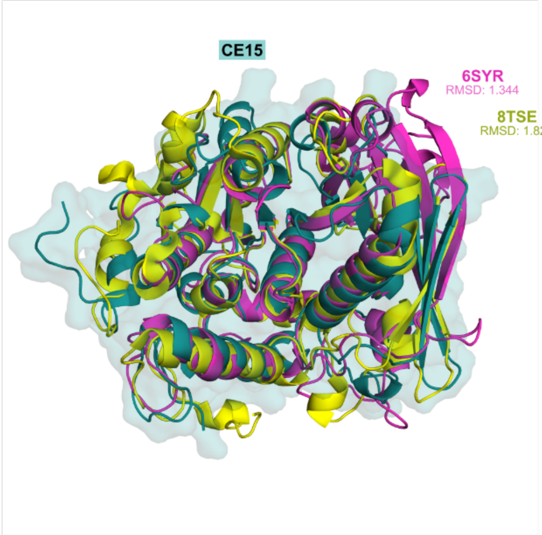

d

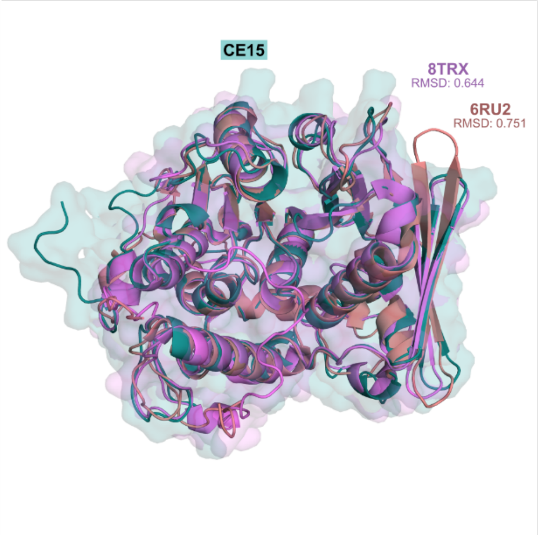

Figure S6. Sequence alignment of homologues of Fl6_CE15. (**a**) Sequences of homologous enzymes from the Protein Data Bank (PDB)[1] were identified using NCBI BLAST®[2,3]. All sequences are characterized members of the CE15 family based on the CAZy database[7]. Sequence alignments were performed using Tcoffee[4] and amino acids were coloured in correspondence to their conservation score. The catalytic triad residues are highlighted with red asterisks. Fl6_CE15 was set as reference for the numbering, which is corresponding to the sequences of native full-length Fl6 (P162_RS02365, AA 49-418). (**b**) Query coverage and percent identify of the sequences with Fl6 and Fl6_CE15 showed no sequence similarity to the characterized CE3 members. (**c**) Structural alignment with the structure of CE15 members 6SYR and 8TSE. (**d**) Structural alignment of the AlphaFold3 model of Fl6_CE15 with CE15 members 8TRX and 6RU2 shows high similarity to Fl6_CE15. Only the esterase domain of each protein is visualized.

a

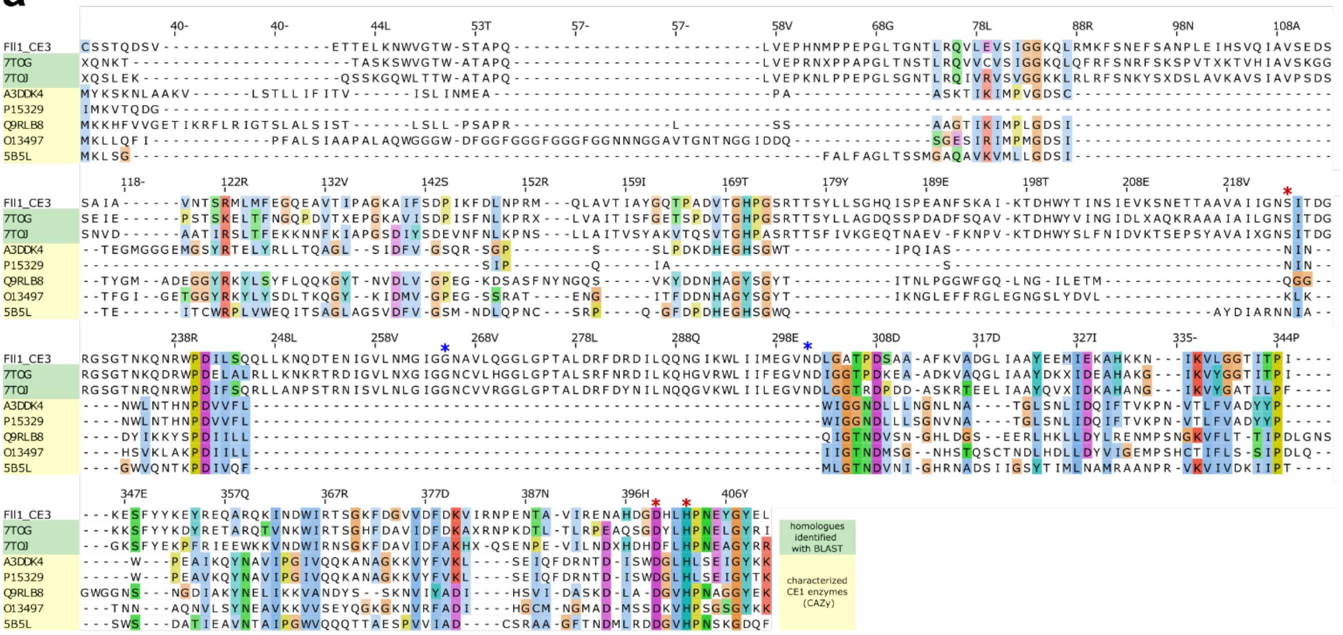

b

	FII1		FII1_CE3	
	Query Coverage [%]	Percent Identity [%]	Query Coverage [%]	Percent Identity [%]
**7TOG**	43	65.01	98	64.96
**7TOJ**	42	56.68	96	57.02

c

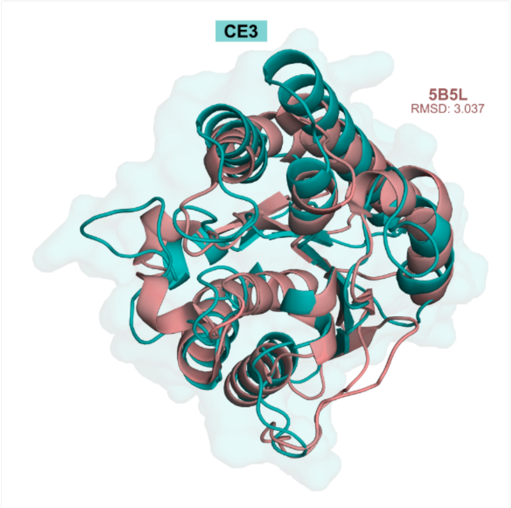

d

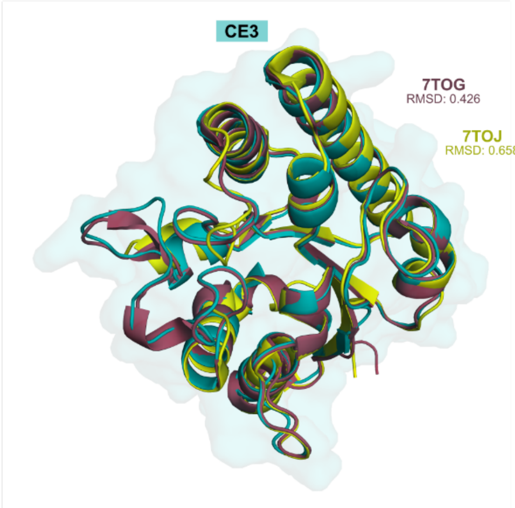

Figure S7. Sequence alignment of homologues of FII1_CE3. (**a**) Sequences of homologous enzymes from the Protein Data Bank (PDB)[1] were identified using NCBI BLAST®[2,3] (green box). Additionally characterized CE3 sequences from the CAZy database [7] were used for alignment (yellow box). Sequence alignments were performed using Tcoffee[4] and amino acids were coloured in correspondence to their conservation score. FII1_CE3 sequence was analysed including the N-terminal unannotated domain. The catalytic triad residues are highlighted with red asterisks. Residues of the oxyanion hole are highlighter with blue asterisks. FII1_CE3 was set as reference for the numbering, which is corresponding to the sequences of native full-length FII1 (P162_RS17810, AA 32-410). (**b**) Query coverage and percent identify of the sequences with FII1 and FII1_CE3 showed no sequence similarity to the characterized CE3 members. (**c**) Structural alignment with the structure of CE3 member 5B5L shows some similarity to FII1_CE3. (**d**) Structural alignment of the AlphaFold3 model of FII1_CE3 with non-classified CEs 7TOG and 7TOJ shows high similarity to FII1_CE3. Only the esterase domain of each protein is visualized.

a

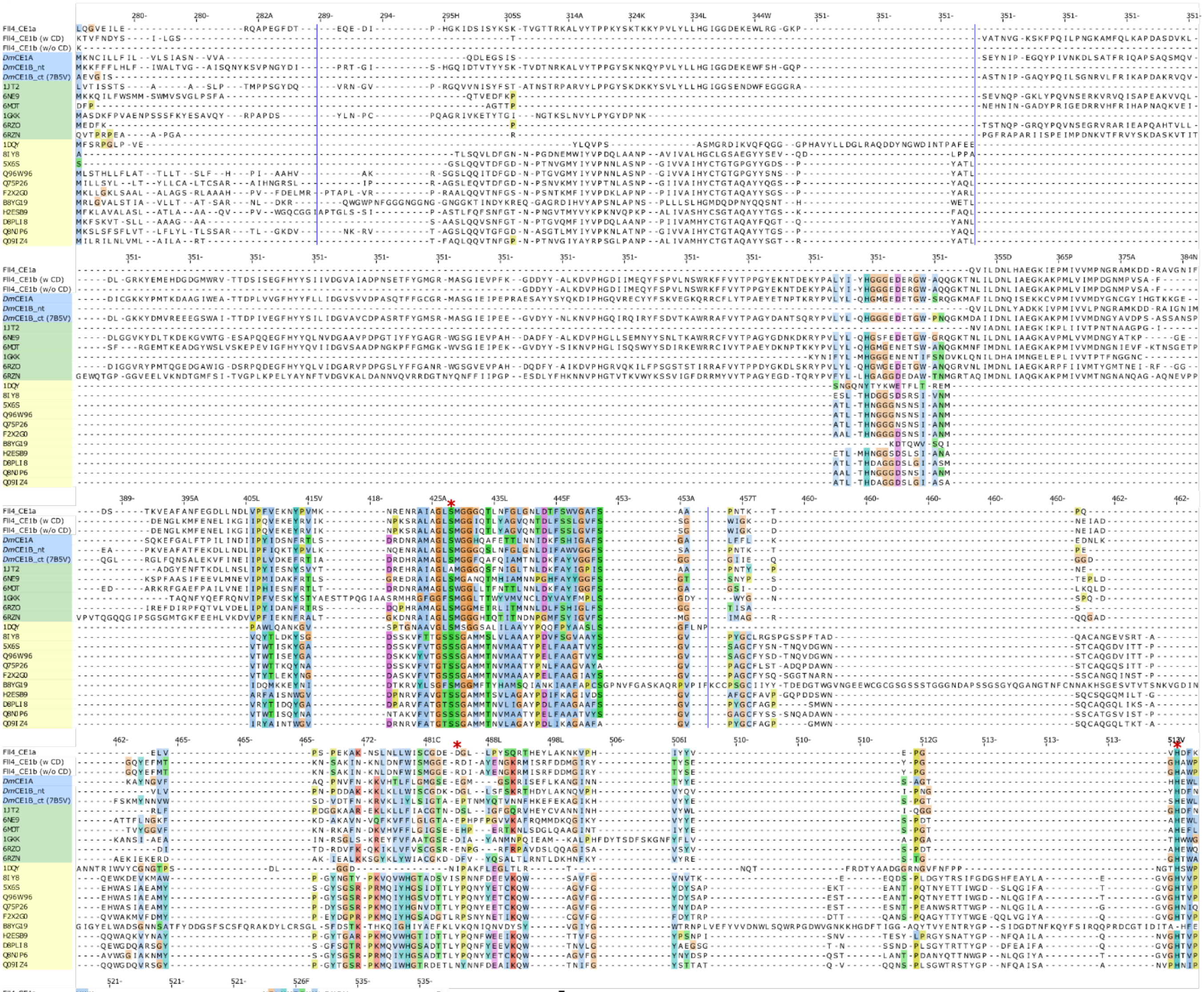

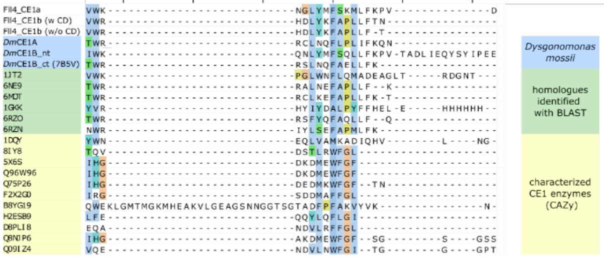

b

	Fll4		Fll4_CE1a		Fll4_CE1b	
	Query Coverage [%]	Percent Identity [%]	Query Coverage [%]	Percent Identity [%]	Query Coverage [%]	Percent Identity [%]
**Fll4_CE1a**	-	-	-	-	67%	39.04
***Dm*CE1B**	78%	55.85	99%	71.09	97%	46.94
***Dm*CE1A**	67%	38.81	98%	37.73	95%	38.81
**6RZO**	66%	37.50	92%	34.82	96%	37.50
**6RZN**	65%	34.24	92%	37.12	93%	34.25
**6NE9**	65%	41.38	92%	33.59	93%	41.38
**6MOT**	67%	41.19	99%	36.13	95%	41.19
**1JT2**	54%	49.02	95%	49.02	65%	34.18
**1GKK**	54%	27.12	77%	27.15	77%	27.12

Figure S8. Sequence alignment of homologues of Fll4_CE1a and Fll4_CE1b. Fll4_CE1b was aligned with (w) and without (w/o) the central, putative CBM48, domain (CD). (**a**) Sequences of homologous enzymes from the Protein Data Bank (PDB)[1] were identified using NCBI BLAST®[2,3] (green box). Additionally esterases with high similarity to Fll4 from *Dysgonomonas mossii*[6] were investigated (blue box) as well as characterized CE1 sequences from the CAZy database[7] (yellow box). Sequence alignments were performed using Tcoffee[4] and amino acids were coloured in correspondence to their conservation score. The catalytic triad residues are highlighted with red asterisks. Large gaps in the alignment, where the sequences of Fll4_CE1a and Fll4_CE1b were interrupted for more than 30 amino acids, were removed for better visualization (blue line). Fll4_CE1a was set as reference for the numbering, which is corresponding to the sequences of native full-length Fll4 (P162_RS04030, AA 272-535). (**b**) Query coverage and percent identify of the sequences with Fll4, Fll4_CE1a and Fll4_CE1b.

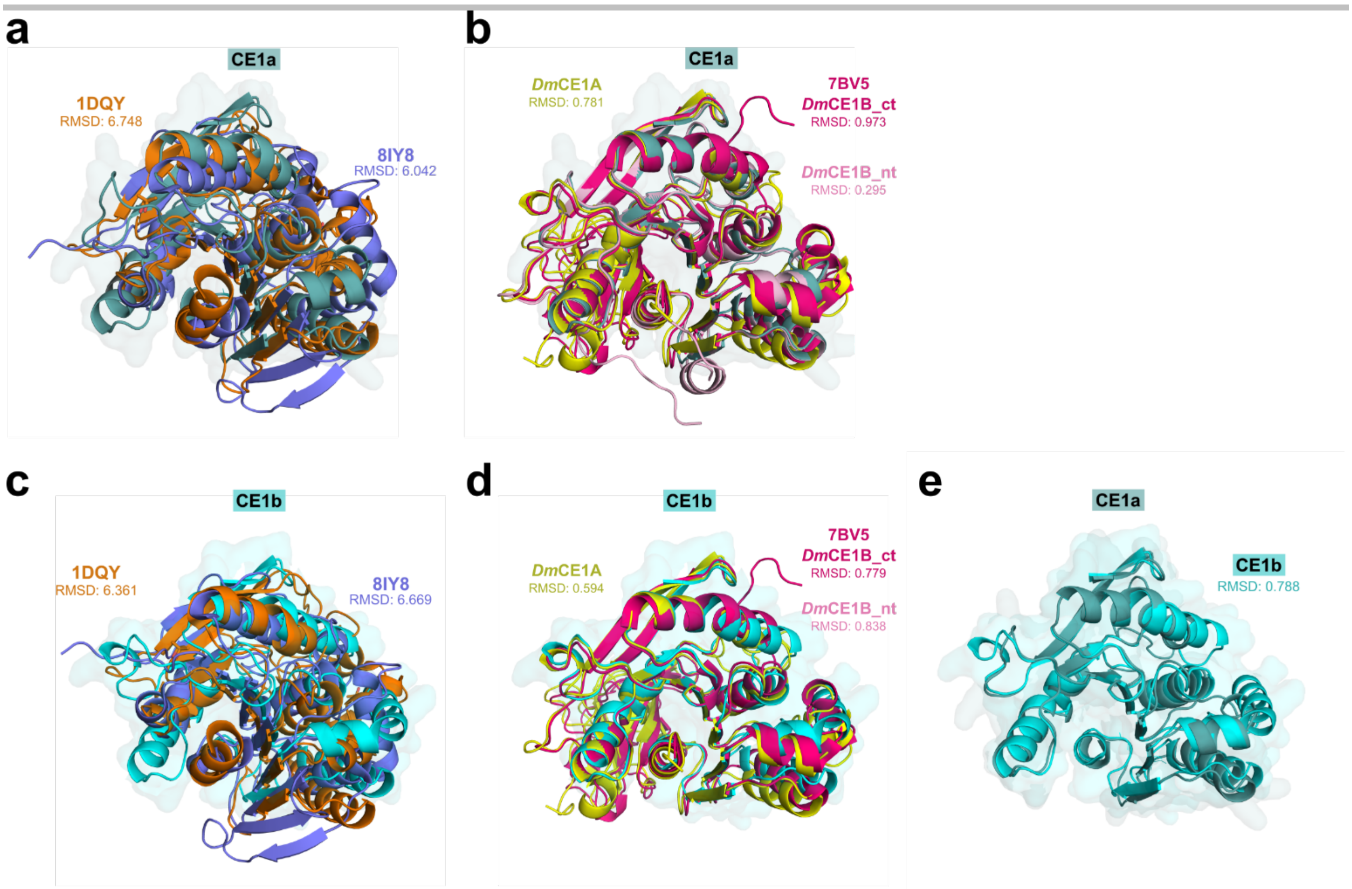

Figure S9. Structural alignments of the AlphaFold3 models of Fll4_CE1a and Fll4_CE1b with selected structures. Only the esterase domains of each protein are visualized. Structures of characterized CE1 family members show little similarity to Fll4_CE1a (**a**) and Fll4_CE1b (**c**), indicated by comparably high RMSD values. Esterases from *Dysgonomonas mossii* (*Dm*) show high structural similarity (RMSD < 1.0) to both Fll4_CE1a (**b**) and Fll4_CE1b (**d**). Even though those enzymes are named CE1, according to CAZy they are assigned to the non-classified CEs, indicating they might belong to a so far unknown CE family. Finally, a superimposition of Fll4_CE1a and Fll4_CE1b also shows high structural similarity of both domains.

a

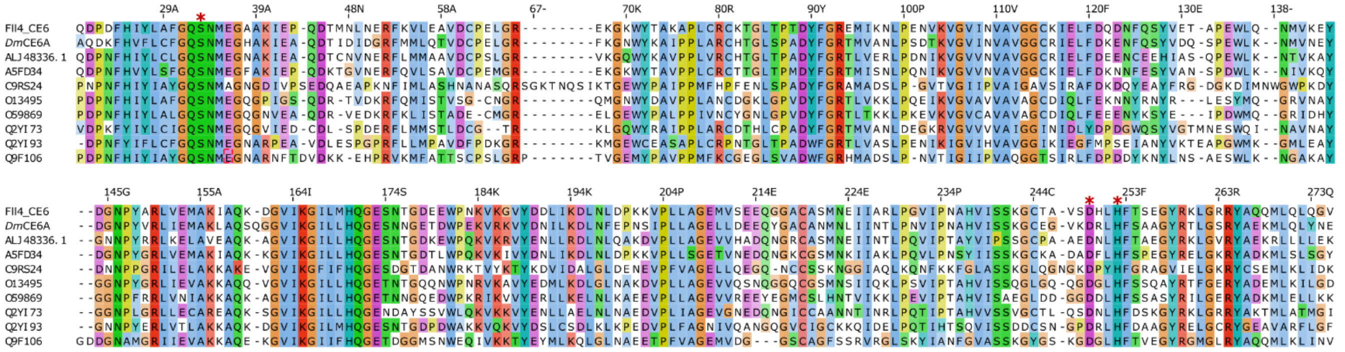

b

	Fll4		Fll4_CE6	
	Query Coverage [%]	Percent Identity [%]	Query Coverage [%]	Percent Identity [%]
**A5FD348**	90	71.64	100	74.90
***Dm*CE6A**	28	68.40	97	68.40
**ALJ48336**	98	67.61	96	67.61
**O59869**	29	54.15	98	54.15
**O13495**	29	54.72	99	54.72
**Q2YI93**	98	51.89	100	52.73
**Q2YI73**	29	54.09	100	53.91
**Q9F106**	33	43.49	100	46.51
**C9RS24**	29	39.02	98	39.02

c

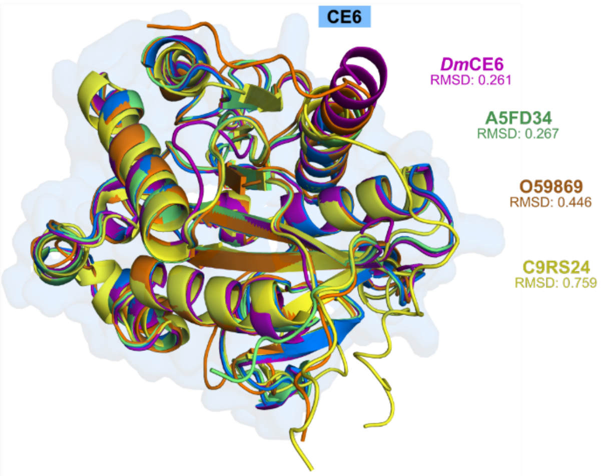

Figure S10. Sequence alignment of homologues of Fll4_CE6. (**a**) Sequences of all characterized CE6 family members from the CAZy database were[7] aligned with the sequence of Fll4_CE6. Sequence alignments were performed using Tcoffee [4] and amino acids were coloured in correspondence to their conservation score. The catalytic triad residues are highlighted with red asterisks. Residues of the oxyanion hole are highlighter with blue asterisks. Fll4_CE6 was set as reference for the numbering, which is corresponding to the sequences of native full-length Fll4. (**b**) Query coverage and percent identify of the sequences with Fll4 and Fll4_CE6. (**c**) Structural alignments of the AlphaFold3 model of Fll4_CE6 with selected models of homologues. No experimentally verified structure is available for characterized CE6 members.[7] Only the esterase domains of each protein are visualized. For the bimodular A5FD34 only the N-terminal SGNH-fold domain is shown.

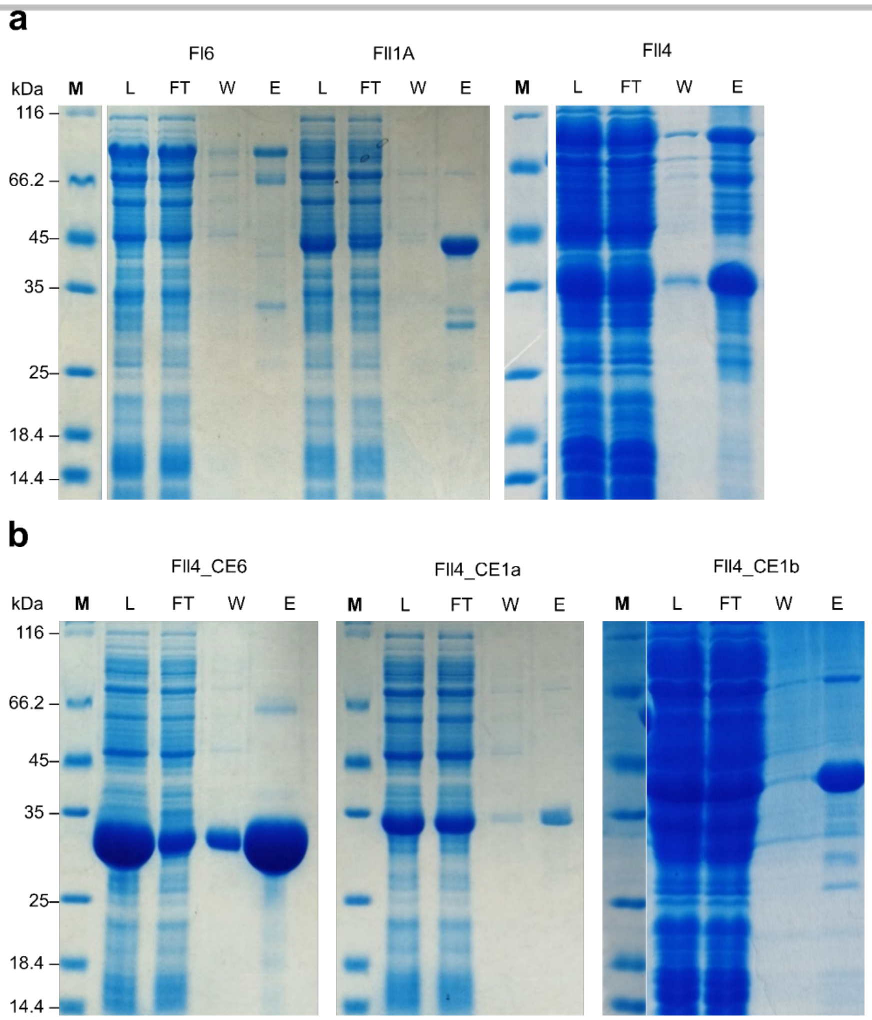

Figure S11: Gravity flow purification of full-length enzymes FI6 (77.6 kDa), FII1_CE3 (43.9 kDa) and FII4 (100.3 kDa) (**a**) and single domains FII4_CE6 (30.8 kDa), FII4_CE1a (31.8 kDa) and FII4_CE1b (42.7 kDa) (**b**). The samples correspond to the fractions collected during the purification process: lysate (L), flowthrough (FT), washing fraction (W) and eluted fraction (E). For reference a protein marker (M) was loaded to each SDS-PAGE. Electrophoresis was carried out at 140V for approx. 1h using a SDS-PAGE containing 12.5% acrylamide.

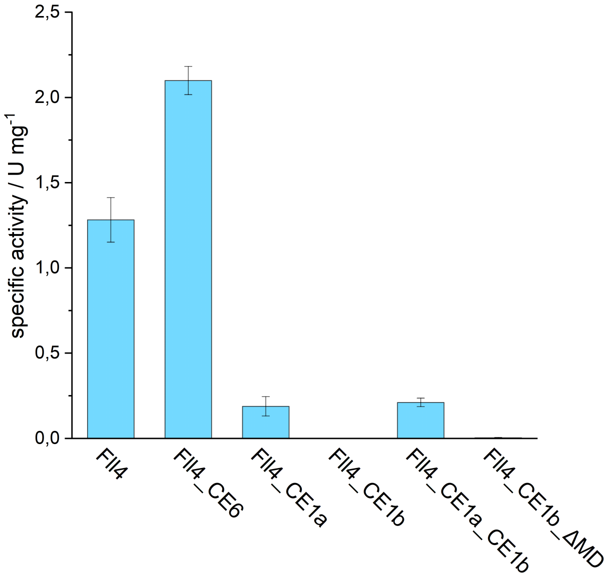

Figure S12: Specific activities of full-length FII4 and its corresponding single domains towards *p*NP-acetate. Additional to the FII4_CE1b two other constructs were tested harboring the CE1a and CE1b domain (FII4_CE1a_CE1b) and one with the CE1b domain without the middle domain (MD) arranged between the CE domains (FII4_CE1b_ΔMD). Activity was determined using final concentrations of 1 mM *p*NP-acetate, 50 mM TRIS-HCl pH 8.0, 100 mM NaCl, 5% DMSO and 25 ug mL^{-1} of the respective enzyme. A negative control without enzyme was measured and subtracted from the values of the enzyme reactions. Mean values and standard deviations were calculated from technical triplicates.

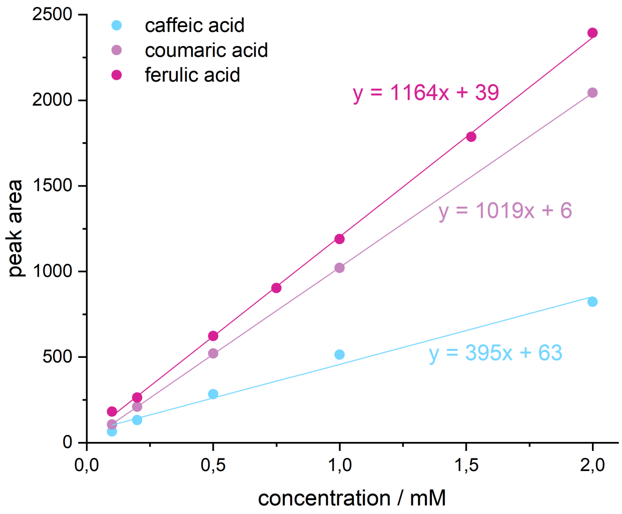

Figure S13: Product standard curves of phenolic acids. Peak areas from HPLC measurements were fitted against the standard concentrations. The linear regression was used for the calculation of the product formation of the enzymatic reactions with phenolic acid esters.

Supporting References

[1] H. M. Berman, J. Westbrook, Z. Feng, G. Gilliland, T. N. Bhat, H. Weissig, I. N. Shindyalov, P. E. Bourne, *Nucl. Acids Res.* **2000**, *28*, 235–242.
[2] S. F. Altschul, W. Gish, W. Miller, E. W. Myers, D. J. Lipman, *J. Mol. Biol.* **1990**, *215*, 403–410.
[3] E. W. Sayers, J. Beck, E. E. Bolton, J. R. Brister, J. Chan, D. C. Comeau, R. Connor, M. DiCuccio, C. M. Farrell, M. Feldgarden, A. M. Fine, K. Funk, E. Hatcher, M. Hoeppner, M. Kane, S. Kannan, K. S. Katz, C. Kelly, W. Klimke, S. Kim, A. Kimchi, M. Landrum, S. Lathrop, Z. Lu, A. Malheiro, A. Marchler-Bauer, T. D. Murphy, L. Phan, A. B. Prasad, S. Pujar, A. Sawyer, E. Schmieder, V. A. Schneider, C. L. Schoch, S. Sharma, F. Thibaud-Nissen, B. W. Trawick, T. Venkatapathi, J. Wang, K. D. Pruitt, S. T. Sherry, *Nucl. Acids Res.* **2024**, *52*, D33–D43.
[4] C. Notredame, D. G. Higgins, J. Heringa, *J. Mol. Biol.* **2000**, *302*, 205–217.
[5] R. J. Gruninger, M. Kevorkova, K. E. Low, D. R. Jones, L. Worrall, T. A. McAllister, D. W. Abbott, *Protein J.* **2024**, *43*, 910–922.
[6] C. Kmezik, S. Mazurkewich, T. Meents, L. S. McKee, A. Idström, M. Armeni, O. Savolainen, G. Brändén, J. Larsbrink, *J. Biol. Chem.* **2021**, *296*, 100500.
[7] E. Drula, M.-L. Garron, S. Dogan, V. Lombard, B. Henrissat, N. Terrapon, *Nucl. Acids Res.* **2022**, *50*, D571–D577.
